# N-substituted phenylbenzamides of the niclosamide chemotype attenuate obesity related changes in high fat diet fed mice

**DOI:** 10.1371/journal.pone.0204605

**Published:** 2018-10-25

**Authors:** Hiral A. Bhagat, Sarah A. Compton, David L. Musso, Christopher P. Laudeman, Kimberly M. P. Jackson, Na Young Yi, Lidia S. Nierobisz, Lawrence Forsberg, Jay E. Brenman, Jonathan Z. Sexton

**Affiliations:** 1 Curl Bio LLC, Durham, North Carolina, United States of America; 2 Biomanufacturing Research Institute and Technology Enterprise, North Carolina Central University, Durham, North Carolina, United States of America; 3 Department of Cell Biology and Physiology, University of North Carolina at Chapel Hill, Chapel Hill, North Carolina, United States of America; 4 Department of Internal Medicine, Division of Gastroenterology and Hepatology, University of Michigan Medical School, Ann Arbor, Michigan, United States of America; 5 Department of Medicinal Chemistry, University of Michigan College of Pharmacy, Ann Arbor, Michigan, United States of America; University of Leeds, Faculty of Medicine and Health, UNITED KINGDOM

## Abstract

Obesity and insulin resistance are primary risk factors for Non-Alcoholic Fatty Liver Disease (NAFLD). NAFLD is generally exhibited by non-progressive simple steatosis. However, a significant subset of patient’s progress to nonalcoholic steatohepatitis (NASH) that is defined by the presence of steatosis, inflammation and hepatocyte injury with fibrosis. Unfortunately, there are no approved therapies for NAFLD or NASH and therefore therapeutic approaches are urgently needed. Niclosamide is an U.S. Food and Drug Administration (FDA)-approved anthelmintic drug that mediates its effect by uncoupling oxidative phosphorylation. Niclosamide and its salt forms, Niclosamide Ethanolamine (NEN), and Niclosamide Piperazine (NPP) have shown efficacy in murine models of diet induced obesity characterized by attenuation of the prominent fatty liver disease phenotype and improved glucose metabolism. While the exact mechanism(s) underlying these changes remains unclear, the ability to uncouple oxidative phosphorylation leading to increased energy expenditure and lipid metabolism or attenuation of PKA mediated glucagon signaling in the liver have been proposed. Unfortunately, niclosamide has very poor water solubility, leading to low oral bioavailability. This, in addition to mitochondrial uncoupling activity and potential genotoxicity have reduced enthusiasm for its clinical use. More recently, salt forms of niclosamide, NEN and NPP, have demonstrated improved oral bioavailability while retaining activity. This suggests that development of safer more effective niclosamide derivatives for the treatment of NAFLD and Type 2 Diabetes may be possible. Herein we explored the ability of a series of N-substituted phenylbenzamide derivatives of the niclosamide salicylanilide chemotype to attenuate hepatic steatosis using a novel phenotypic *in vitro* model of fatty liver and the high fat diet-fed mouse model of diet induced obesity. These studies identified novel compounds with improved pre-clinical properties that attenuate hepatic steatosis *in vitro* and *in vivo*. These compounds with improved drug properties may be useful in alleviating symptoms and protection against disease progression in patients with metabolic syndrome and NAFLD.

## Introduction

Niclosamide was originally developed and marketed as a molluscicide by Bayer in the late 1950s; it was later found to be an effective anti-helmintic and has been used in the clinic for decades to treat intestinal tapeworm infections in humans [1, 2]. Niclosamide exerts its anti-helmintic effect by uncoupling the electron transport chain from ATP synthase preventing the synthesis of ATP, an essential energy molecule for cell metabolism [3, 4]. Historically, the use of mitochondrial uncouplers in humans has been avoided due to their low therapeutic index [5]. Indeed, the best known mitochondrial uncoupler, 2,4-dinitrophenol (DNP) increases metabolic rate and stimulates loss of body fat but has been associated with severe toxicity including fatal increases in body temperature [5–7]. This side effect may be overcome by liver specific targeting of DNP which has been shown to improve its therapeutic index up to 50-fold [8]. In contrast to DNP, niclosamide has been shown to be relatively safe when delivered as a single 2-gram oral dose to treat intestinal cestode infections. This is largely due to its poor absorption from the gastrointestinal tract and high plasma protein binding that significantly limit its tissue distribution [1]. On the other hand, this also limits the usefulness of niclosamide to treat other diseases where systemic exposure is required. Several other studies have linked niclosamide treatment to genotoxic effects as measured by increased sister chromatid exchange (SCE) and chromosome aberrations (CA) in bone marrow cells or sperm head abnormalities in mice [9, 10]. Niclosamide also generates point mutations in *Salmonella sp* and clastogenic effects in human lymphocytes [9, 10]. Therefore, while niclosamide has been shown to be relatively safe after a single dose, it is unclear if chronic treatment with niclosamide would lead to adverse effects.

Despite possible concerns over bioavailability and genotoxicity, interest in niclosamide has risen dramatically over recent years, concomitant with the movement towards repurposing approved drugs. Indeed, niclosamide has been identified in several screens as a compound with broad anti-cancer activity. This has been attributed to its ability to regulate multiple cellular signaling pathways frequently dysregulated in cancer biology including nuclear factor-κB, JAK1-STAT3, Wnt/Beta Catenin, Wnt/Frizzeld1, mTORC1/AMPK and NOTCH [11–17]. Niclosamide has also been proposed as a treatment of a variety of other indications including neuropathic pain, bacterial and viral infections and metabolic disease [11, 14, 18–22]. We also identified niclosamide in a high throughput screen focused on identifying modulators of non-classical peroxisome biogenesis with potential for alleviating the symptoms of dyslipidemia and metabolic syndrome [23]. Two salt forms of niclosamide with improved solubility, Niclosamide ethanolamine salt (NEN) and Niclosamide Piperazine (NPP) have been shown to attenuate hepatic steatosis and glucose metabolism in murine models of high fat diet (HFD) induced obesity and Type 2 Diabetes (T2D) [3, 23, 24]. However, the mechanism for niclosamide’s beneficial effect on hepatic steatosis and whole-body glucose metabolism remains unclear. Such effects may be mediated by mild mitochondrial uncoupling, increased energy expenditure and increased lipid metabolism, or the ability of niclosamide and NEN to block hepatic glucagon signaling pathway [3, 25]. Regardless of the underlying mechanism, when compared to niclosamide, NEN and NPP have improved oral bioavailability and retain activity in mouse models of T2D and metabolic disease [3, 24]. Moreover, despite their mild mitochondrial uncoupling activity, both are well tolerated *in vivo* and do not appear to exert any adverse effects on body temperature [3]. To our knowledge, the effect of these salt forms of niclosamide on genotoxicity have not been reported, however, we anticipate that both salts would be similarly genotoxic as niclosamide.

Given the dramatic effects of niclosamide on hepatic steatosis and glucose metabolism there is significant interest and opportunity to develop novel niclosamide derivatives with improved efficacy and translational potential. Several new niclosamide derivatives have been described that have divergent activities on ATP homeostasis and the Wnt pathway suggesting that niclosamide derivatives that lack uncoupling activity can be made with improved pharmacological properties to treat different indications [26]. Herein we explore a series of phenylbenzamide derivatives of the salicylanilide chemotype and evaluate their potential as NAFLD therapeutics using *in vitro* and *in vivo* models of hepatic steatosis, obesity and T2D.

## Materials and methods

### General chemistry procedures

The purity of all of the benzamides used in this study was confirmed by HPLC (Agilent Technologies 1200 series) and found to be >96% pure. The structures were confirmed by mass spectrometry (Agilent Technologies 6130 Quadrupole) and ^1^H-NMR (Varian 500 MHz) and found to be consistent with the desired products. Commercially available reagents for the synthesis described below were used as received. A full description of the synthetic protocol for all molecules described herein is available in S1 supporting information.

### Cell culture conditions

Non-neoplastic human PH5CH8 hepatocyte cells were kindly provided by Stanley M. Lemon, University of North Carolina Chapel Hill and maintained in Dulbecco’s Modified Eagle’s Medium (DMEM) containing 4500 mg/L glucose and L-glutamine (Life Technologies) supplemented with 10% Fetal Bovine Serum (FBS), 1X penicillin-streptomycin (Thermo Fisher Scientific) and 1 mM sodium pyruvate (Thermo Fisher Scientific) as previously described [27]. PH5CH8 cells are not commercially available and Short Tandem Repeat (STR) profiles are not in existing databases. Therefore, PH5CH8 cells were authenticated using STR profiling (Genetica) and compared to reference databases (ATCC, DSMZ and Cellasuarus) to exclude contamination with known cell lines. Human embryonic kidney (HEK) 293T cells were obtained from ATCC (Manassas, VA) and maintained in complete DMEM (Life Technologies; Thermo Fisher Scientific) containing 10% FBS (Atlanta Biologicals) at 37°C in 5% CO_2_. Human liver HEP-G2 cells (ATCC HB-8065) were also obtained from ATCC and maintained in complete DMEM containing 10% fetal bovine serum at 37°C at 5% CO_2_.

### High -throughput hepatic *de novo* lipogenesis (DNL) assay

The hepatic de novo lipogenesis (DNL) assay was developed in 96-well format using Poly-D-lysine coated 96-well black optical bottom plates with polymer base (Greiner Bio-One #655946). PH5CH8 cells were harvested and counted using a Vi-Cell XR (Beckman-Coulter) and seeded at 3000 cells per well in 200 μl of PH5CH8 culture media. After 48 h, the media was removed and replaced with 200 μl of fresh culture media. A BioMek NX workstation using a 96-multichannel head was used for compound addition. Compounds were tested in quadruplicate with concentrations ranging from 20 to 0.039 μM. Compounds were first diluted in 10-point 2-fold format in a 96-well plate and 1μl of each compound or DMSO was directly dispensed to each well of the cell plate, thereby avoiding a serial dilution step. The final DMSO concentration was 0.2%. After 4 hours, 20 μl of media with compound was removed from each well using the Biomek NX workstation. Except for the minimum signal control which received 20 μl of DMEM, all wells received 20 μl a 10 mM stock solution containing a mixture of insulin and LXR agonist T0901317 prepared in DMEM to each well to give a final concentration of 1 μM insulin and 1 μM T0901317. Cells were cultured for an additional 72 hours to induce *de novo* lipid synthesis. Upon completion, the media was removed from each well using a multichannel pipette and the cells were then washed once with phosphate- buffered saline (PBS) prior to fixation and staining.

Immediately following the PBS wash step, 100μl of 10% Neutral-Buffered Formalin (NBF) containing 10 μg/ml cell-permanent nuclear stain, Hoechst-33342 was added to each well and incubated at 37°C with 5% CO_2_ for 15 minutes. The cells were then washed once with 100 μl PBS and HCS LipidTOX green neutral lipid stain (Thermosfisher) was applied following a 1:1000 dilution in PBS. Cells were incubated with the LipidTOX stain at room temperature for 30 min. The plates were sealed with aluminum foil using a Thermo-ABgene plate sealer and imaged using a CellInsight CX5 High Content Screening (HCS) Platform within 2 hours of processing.

### DNL assay image acquisition and data analysis

A 20X/0.45NA Olympus LUCPlanFLN microscope objective was used with 2-fold camera binning, leading to 0.2μm per pixel resolution. The LipidTOX green stain used to demarcate lipid content was excited using a 485/20 nm excitation LED. Hoechst signal (nucleic acid) was excited using a 386/23 nm LED. Image analysis was performed using the open source Cell Profiler software and used for cell identification, feature extraction and data tabulation [28]. A two-pass (high/low) thresholding process was used to identify both the nuclei and cytoplasm in the Hoechst-33342 channel and a pipeline was developed to automatically identify nuclei, cytoplasm and for feature extraction. Cell level data was analyzed in JMP12 (SAS, Cary NC). Hundreds of primary features per cell were generated based on the intensity measurements and morphological characteristics. We employed a Partial Least Squares (PLS) regression analysis method to identify the most important cellular features and reduced the data set to a single scoring system that is tolerant to outliers for hepatic steatosis [29]. The typical steatotic “fingerprint” score represents approximately 45 individual features including lipid droplet count, intensity, size and cellular morphology. Data were normalized to percent control (NPC) using the equation NPC = (X_i-_—C_-_)/(C_+_—C_-_) x100, where X_i_ is the raw value, C_+_ plate mean of positive control, C_-_ plate mean of negative control [30]. The values were averaged and compounds were identified as positive hits if they reduced steatotic phenotype by more than 45% relative to maximum signal control and they were dose responsive. Z′-values were calculated using Z′ = 1- [3(σ_p_+σ_n_)/(μ_p_-μ_n_)] where μ is the mean and σ is the standard deviation of (p) positive and (n) negative controls.

### Measurement of cellular respiration

Oxygen consumption rate experiments were performed by ZenBio, Inc (RTP, NC). HEK293 cells stably transduced with the human OCT3 transporter were plated in black-walled microclear plates (Greiner-BioOne) at 60,000 cells per well in growth medium. Cells were incubated overnight to allow attachment before growth medium was removed and replaced with 150μL of assay medium containing compound dilutions. Four concentrations of each compound were prepared and transferred to the empty wells. Control wells contained assay medium only or assay medium containing p-trifluoromethoxyphenylhydrazone (FCCP) at 1μM. Ten μL of MitoXpress Xtra reagent was added to each test well and 100μL of pre-warmed mineral oil was overlaid in each well to create a seal. The plates were immediately read on a FLUOstar Omega (BMG) using dual read Time Resolved Fluorescence (TR-F) for 2 hours, with measurements taken every 2 minutes using 340nm and 650nm as excitation and emission wavelengths respectively. For lifetime analysis, two measurements per well were taken: 1) delay of 30μs, with a window of 30μs; and 2) delay of 70μs with a window of 30μs. Background TRF values were subtracted from all readings. A ratiometric analysis was performed to determine changes in the Lifetime fluorescence signal: Lifetime (μs)[T] = (D2-D1)/ln(W1/W2), where D is delay; W is fluorescence window value at each time point. Lifetime slopes were calculated using each Lifetime measurement over time. Data are presented as mean ± standard error (SE). The differences between the control (DMSO) and compound treated wells were analyzed using the Student’s t-test. A value of p≤0.05 was considered statistically significant.

### Protein extraction and immunoblotting

HEK293T cells were seeded in 6 well plates and cultured to 50–60% confluence. Cells were fed new media 24 hours before the addition of compounds to reduce basal AMPK phosphorylation. Compounds were then delivered in fresh media for 3 hours before harvesting cells for Western blot analysis. Protein lysates for immunoblotting were prepared by suspending cell pellets in lysis buffer (50 mM Tris/HCl, pH 7.5, protease and phosphatase inhibitor cocktails (Sigma–Aldrich) and benzonase nuclease plus 1.0% Triton X-100) with shaking for 1 hour (4°C) followed by centrifugation at 16,000 *g* for 10 min (4°C) in 1.5-ml microfuge tubes. Supernatants were collected and protein concentrations were determined using the Bio-Rad DC protein assay. Proteins (50 μg) were then boiled in loading buffer and subjected to SDS/PAGE (Invitrogen), followed by Western analyses using the following primary antibodies: phospho-AMP activated protein kinase (AMPK) (Thr 172; Cell Signaling #2535), total AMPK (Cell Signaling #2603), Fatty acid synthase (FAS; Cell Signaling #3180) and Carbonic Anhydrase 3 (CAR3; Santa Cruz Biotechnology #sc-50715). Secondary antibodies (IRDye infrared antibodies; LI-COR Biosciences (Lincoln, NE); anti-Goat IgG #926–32214, anti-Rabbit IgG #926–68073) were used at a dilution of 1:2000. Scanning, analyzing and quantification of blots were performed using the Odyssey Infrared Imaging System (LI-COR Biosciences). Western blotting of mouse liver tissue lysates was performed as described previously [31].

### High-throughput phosphorylated AMPK activation assay

Phosphorylated AMPK (Thr-172) TR-FRET Assay Kit was purchased from CisBio (Bedford MA). HepG2 [HB-8065] cells were trypsinized, harvested and counted using Countess II FL automated cell counter (Thermo Fisher Scientific). Cells were then seeded in Poly-D-Lysine coated 96-well black optical plates at a concentration of 10,000 cells per well in 50μL growth medium. After 24-hours, the media was removed and replaced with 50μL of fresh growth media containing compounds. Five concentrations of each compound were prepared, ranging from 10 to 0.625 μM and applied to columns 2–11 of the cell plate. Control wells contained DMSO (column 1-minimum response control) or 500199 (column 12-maximum response control). The final DMSO concentration was 0.45%. Cells were cultured for 4 hours before cells were lysed in 50μL of lysis buffer for 30 minutes at room temperature according to assays protocol. Following lysis, 16μL of lysate was transferred to white 384 well small volume assay plate and 4μL of antibody dyes were added per assay protocol.

Following overnight incubation, quantitation was performed on a PHERAstar (BMG), using the HTRF protocol with an excitation wavelength of 337nm and emission wavelengths of 665nm and 620nm. The ratio between emission spectra (665/620) was assessed for each well individually and averaged for triplicate samples. Data are presented as mean ratio ± SE. The differences between the control (DMSO) and the compound treated wells were analyzed using two-way ANOVA and Dunnett’s post hoc test. A value of p<0.05 was considered to be significant.

### Metabolic stability in human and mouse liver microsomes

Human and mouse liver microsomes were purchased from Corning (lot numbers 38291 and 5139008 respectively). Three experiments were performed with 1) 0.5mg/ml microsomes and 1mM NADPH, 2) 0.5mg/ml microsomes without NADPH and 3) 0.5mg/ml heat inactivated microsomes without NADPH in 100mM phosphate buffer, containing 5mM magnesium chloride. The reactions were initiated by the addition of 200μM experimental compound or 2μM verapamil (control) and incubated at 37°C. At the indicated time points, 50μl samples were removed and stopped by addition of 4 volumes of cold acetonitrile with internal standards, 100nM alprazolam, 200nM labetalol and 2μM ketoprofen. The samples were centrifuged at 3,220 g for 40 minutes and aliquots of 100μl were subsequently diluted with 100μl of ultra-pure water and analyzed by LC/MS/MS using a (Shimadzu corporation) LC system coupled to a Shimadzu Triple Quad 5500 mass spectrometer with an ESI interface. The LC system used a Waters XSelect HSS T3 C18, 2.5μm, 2.1 x 50 mm column eluted with one of two methods using mobile phases A (water, 0.1% formic acid) and B (acetonitrile, 0.1% formic acid). **Method 1**: Injection volume: 1 μL; Elution rate: 0.6 mL/min; Gradient: 0–0.3 min (5%B), 0.3–0.8 min (5–100%B), 0.8–1.2 min (100%B), 1.2–1.5 min (100–5%B), 1.5–2.0 min (5%B). **Method 2**: Injection volume: 1 μL; Elution rate: 0.6 mL/min; Gradient: 0–0.3 min (20%B), 0.3–0.8 min (20–100%B), 0.8–1.2 min (100%B), 1.2–1.5 min (100–20%B), 1.5–2.0 min (20%B). The Shimadzu Triple Quad 5500 MS was operated with the following parameters: Ion source: Turbo spray; Ionization mode: ESI; Scan type: MRM; Collision gas: 10 L/min; Curtain gas: 30 L/min; Nebulize gas: 50 L/min; Auxiliary gas: 50 L/min; Temperature: 500°C; Ionspray voltage: +5500 V (positive MRM). All experiments were performed in duplicate. Data were analyzed using Microsoft excel, and peak areas were determined from extracted ion chromatograms.

### Pharmacokinetic profile in Sprague Dawley rats

To evaluate the pharmacokinetic profile by oral (PO) and intravenous (IV) administration routes we used 6 to 8-week old Sprague Dawley rats weighing approximately 200-330g (n = 3 per study group). Compounds were administered at 1mg/kg in 5% Cremophor EL and 20% PEG400 in water for IV and 5mg/kg in PBS for PO. All dose formulation was performed at room temperature. Animals were fasted overnight prior to dosing and were re-fed approximately 4 hours after dosing. For PO, 0.2ml of blood was collected pre- and 0.5, 1, 2, 4 8 12 and 24-hours after drug delivery and transferred to EDTA-K2 BD Vacutainer plastic blood collection tubes. IV samples were collected pre-injection and 0.08, 0.25, 0.5, 1, 2, 4, 8, 12, 24-hours post injection. Blood samples were centrifuged at 2,000 g for 5 minutes at 4°C to obtain plasma. Plasma samples were stored at -75±15°C prior to analysis and then thawed and analyzed by LC-MS/MS method and pharmacokinetic calculations were performed using WinNolin (Phoenix, version 6.4). For IV administration: T_1/2_, C_0_, AUC_last_, AUC_inf_, MRT, Cl, Vss, and number of points for regression were determined. For PO administration: T_1/2_, C_max_, T_max_, AUC_inf_, AUC_last_, F_(0-∞)_ %, and number of points for regression were determined. Data are reported as mean and standard deviation.

### Genotoxicity

The Ames assay was used to evaluate genotoxicity. Strains of S*almonella typhimurium* (TA98, TA100, TA1535 and TA1537) and the tryptophan auxotrophic strain of *Escherichia coli* (WP2 uvrA (pKM101)) were used to detect reverse mutations at the histidine locus in the presence or absence of exogenous metabolic activation (Aroclor 1254 induced rat liver S9). The assay was conducted in the presence or absence of the S9 mixture along with the negative control (DMSO) and positive controls. The positive control chemicals were 2-Aminoanthracene (2-AA) for all S9 activation experiments, 2-nitrofluorene (2-NF) for TA98, Sodium Azide (SA) for TA100 and TA1535, ICR-191 for TA-1537 and N-methyl-N′-nitro-N-nitrosoguanidine (MNNG) for WP2 uvrA. Compounds were tested at 1.5, 4, 10, 25, 64, 160, 400 and 1000 μg /well. Compounds were considered positive if they induced ≥3-fold increases for the mean number of revertant colonies compared to the negative control (DMSO).

### Animals and diets

Rodent studies described in this manuscript were approved by the North Carolina Central University's Institutional Animal Care and Use Committee under protocol number JZS-04-24-2013. Mice were purchased from Jackson Laboratory (Bar Harbor, ME) and were singly housed at constant temperature and light/dark cycles of 12 hours a day. Animals were cared for in an Assessment and Accreditation of Laboratory Animal Care (AALAC)-Accredited facility at Biomedical Biotechnology Research Institute of North Carolina Central University. All experimental protocols were approved by the Institutional Animal Care and Use Committee. Fifty-four 19-week old male C57BL6 diet induce obesity (DIO) mice were allowed to acclimatize for 1 week prior to initiation of the study. Mice had access to water and 60 kcal % fat diet (Research Diets, D12492i) *ad libitum*. Animals were injected once daily by intraperitoneal (IP) 25μL injection with a solution of DMSO alone or compounds at the indicated doses dissolved in DMSO as previously described [31]. Six wildtype C57BL/6 mice fed standard chow diet were used as controls and remained untreated.

### Mouse liver histopathology and quantitative histomorphometric analysis

After 12 days of treatment, mice were humanely euthanized, the entire liver was excised and liver color was immediately evaluated. Liver tissues were collected from the lower portion of the left lobe and formalin fixed. Formalin-fixed, paraffin-embedded liver tissues were sectioned at a thickness of 8μm, then stained with hematoxylin and eosin (H&E) and used to evaluate changes in liver histology. The H&E stained liver sections were scanned at 400X total magnification using an Aperio ScanScope slide scanner. Histological examination was performed using the standardized histological scoring system for NAFLD [32]. Degrees of steatosis were graded as follow: ≤ 5%:0, 5–33%:1, 33–66%:2, 66–100%:3. Fatty infiltration was classified as microvesicular, macrovesicular or mixed, and the location (Zone 1–3 or azonal) was recorded. For image analysis, we used automated steatosis phenotyping using a custom image-processing algorithm and data analysis pipeline created using Cell Profiler software. This algorithm automatically identified and analyzed macro- and micro vesicular steatosis based on the size, shape, and location of lipid droplets and intensity/texture of cytoplasmic staining, respectively. We then analyzed the data using Partial Least Squares (PLS) regression analysis to generate a multivariate scoring system with JMP12 software (SAS, Cary NC). The PLS scoring system takes the tissue average macrosteatosis and microsteatosis scores normalized to tissue area and multiplied by their respective importance factors and sums them into an overall score for hepatic steatosis using the sham animals as controls (steatosis score of 1.0).

### Statistical analysis

Statistical analysis was performed using JMP 12 and GraphPad Prism7. Data are presented as mean ± SE. The differences between the sham and compound treated groups were analyzed using the Student’s t-test. A value of P≤0.05 was considered statistically significant.

## Results

### A hepatic *de novo* lipogenesis (DNL) assay for identification of NAFLD active compounds

*De novo* lipogenesis (DNL) is an important contributor to the pathogenesis of NAFLD and is abnormally elevated at a rate 3-fold higher in patients with this condition compared to body mass index matched controls [33–35]. It is also upregulated in people with metabolic disorders including T2D and obesity [36–40]. DNL is governed by a variety of lipogenic transcription factors including liver X receptors (LXR), sterol regulatory element-binding protein-1c (SREBP-1c) and carbohydrate response element binding protein (ChREBP)[41, 42]. In the liver, transcription of SREBP-1c is activated by insulin and leads to the activation of genes involved in fatty acid and triglyceride synthesis. LXRs also regulate lipogenic genes and are required for the transcriptional control of SREBP-1c by insulin [41].

**Fig 1:**
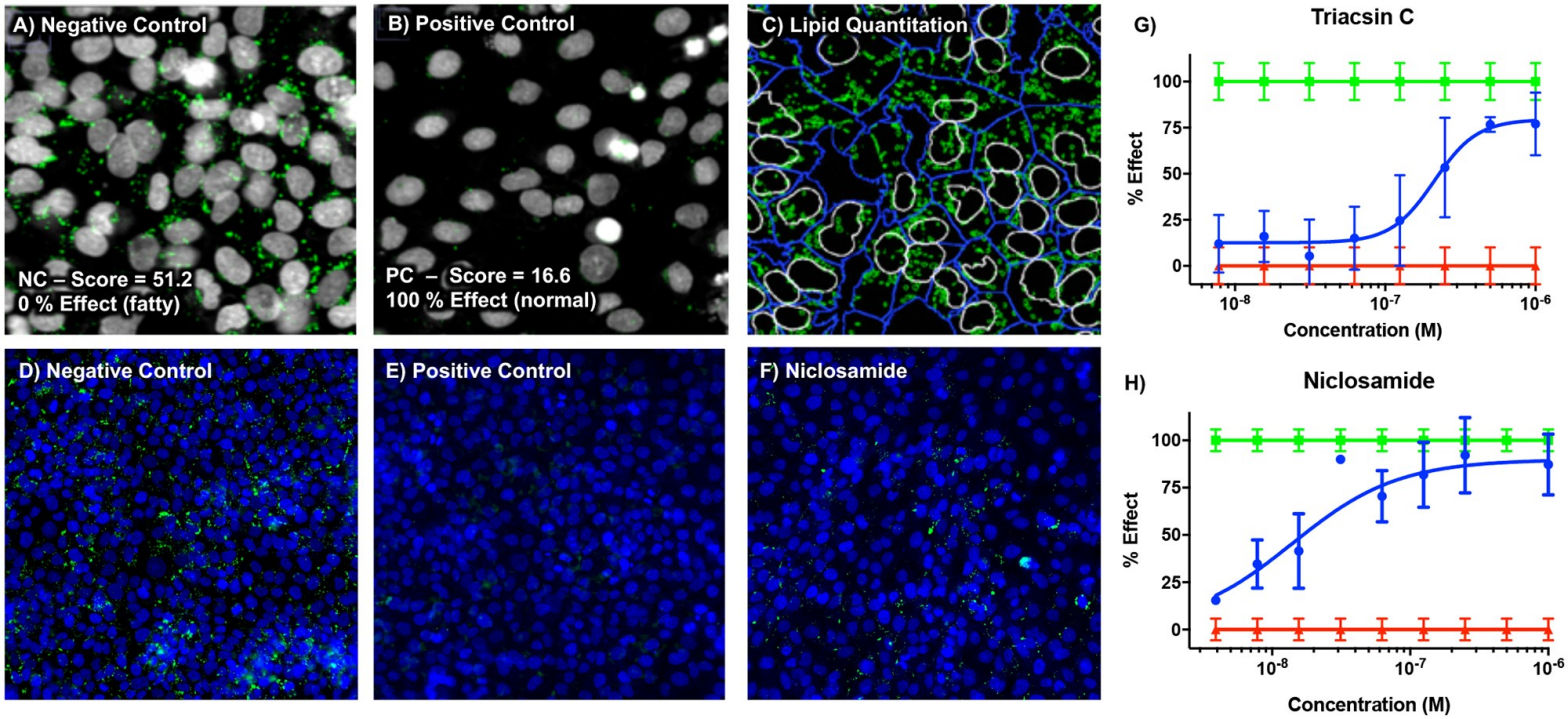
High content lipid quantitation in PH5CH8 liver cells. Negative control cells stimulated for *de novo* lipid synthesis showing nuclei (gray) and lipids (green) (A) and lipid quantitation of negative control using high-content image analysis resulting in a high lipid score, normalized to 0% effect (C) Positive control—unstimulated cells representing the normal (basal) lipid content with a low lipid score and normalized to 100% effect (B). Representative images from high throughput screening showing negative (D) and positive (E) control wells and stimulated cells treated with 1 μM niclosamide (F). Dose response analysis of test compounds Triacsin C (G) and niclosamide (H) shown in blue. Green and red lines represent the mean and standard error of stimulated and unstimulated cells.

Based on previous studies showing the effects of niclosamide and its salt forms NEN and NPP on hepatic steatosis, we sought to establish a cell-based disease model of NAFLD using insulin and the LXR agonist T0901317 to stimulate *de novo* hepatic lipid accumulation to study the effects of related N-substituted Phenylbenzamides on steatosis in a non-neoplastic human hepatocyte cell line, PH5CH8 [27]. Following stimulation and lipid accumulation, cells were fixed and labeled with HCS LipidTOX green neutral lipid stain and the cell-permeant nuclear counterstain Hoechst-33342. Images of labeled cells were captured on a Cellomics/Thermofisher CX5 automated high-content microscope using an Olympus 20X LWD/0.7NA objective. Images of unstimulated and stimulated cells were collected and automated image analysis was performed to tabulate the cellular lipid content (green channel) and total cell count (blue channel). Fig 1A–1C shows the negative controls stimulated with 1 μM insulin and 1 μM T0901317 for 72 hours and positive controls (no stimulation) and the resulting segmentation and identification of nuclei (white lines), cells (blue lines) and lipid droplets (green lines) using high-content image analysis. The unstimulated positive control shown in Fig 1B and 1E represents the basal lipid content for PH5CH8 cells. The insulin and T0901317 stimulated negative control cells accumulated significant lipid droplets and developed the steatotic phenotype as shown in Fig 1A and 1D. This phenotype was ameliorated by niclosamide as demonstrated by the lipid droplet accumulation in panel F.

We further developed a custom image-processing algorithm and data analysis pipeline based on the open source Cell Profiler software platform. This enabled accurate quantitation of the steatotic phenotype and key cellular features including the identification of nuclei, cell boundaries, and lipid droplets contained therein. Once these regions were accurately identified, features were extracted from each cell for a combination of response factors. The data output for this assay generated hundreds of primary features per cell including intensity measurements, morphological characteristics and subcellular features that can be aggregated as a “phenotypic fingerprint” of the steatotic phenotype.

From all of the features extracted, we have identified the 45 most important cellular features that change in response to lipid accumulation including cell number, total lipid intensity, lipid spot count and lipid spot size. We used Partial Least Squares (PLS) regression analysis to generate a multivariate scoring system for these most critical features. The ultimate goal was data reduction towards a single robust scoring system that captures the steatotic phenotype and that is tolerant to the outlier effects. The PLS scoring system takes the well-averaged features multiplied by their respective importance factors and sums them into an overall score for the well. The score is normalized per-plate to 0–100% effect using the “unstimulated control” (100% Effect) and “stimulated + DMSO Vehicle” (0% effect) as controls. This bio-imaging platform consistently yielded an average Z-prime factor above 0.49. Moreover, the assay includes an internal measure to evaluate *in vitro* liver toxicity based on the nuclei counting feature using the Hoechst channel. Compounds were considered toxic if the cell count fell below 80% of the vehicle control values for cell number.

In an effort to validate this phenotypic assay and to better understand the clinical relevance of the DNL assay, we tested niclosamide, Metformin—the classic anti-diabetic drug, and Triacsin C—a potent inhibitor of long fatty acyl CoA synthetase for their ability to attenuate the steatosis phenotype in this platform. Triacsin C and niclosamide both inhibited the steatotic phenotype in PH5CH8 in a concentration dependent manner, albeit with different potencies (Fig 1G and 1H). Triacsin C and niclosamide reduced steatosis by 92 ± 19% and 77 ±12% respectively.

We concluded that the resulting high content imaging, cell-based disease model of hepatic steatosis provides a novel method to evaluate compounds for their ability to improve hepatic steatosis either by suppression of *de novo* lipid synthesis or stimulating downstream catabolic pathways, including fatty acid oxidation. This cell-based efficacy assay detects therapeutically-relevant improvements in metabolic phenotype for cell-permeable compounds and aids in the prioritization of compounds for further development as potential therapeutics.

### Inhibition of *in vitro* hepatic steatosis by phenylbenzamides

Based on the DNL assay results, we used this platform to systematically explore the phenylbenzamide chemotype with the goal of identifying compounds that retained the robust hepatic lipid lowering activities but with improved drug-like properties.

As previously mentioned, the nitro group on niclosamide imparts poor solubility, limits oral bioavailability and is a potentially liver-toxic moiety, so our initial goal was to improve absorption characteristics of experimental compounds by replacing the aromatic nitro group with an electron withdrawing ester or acid moiety [43]. Furthermore, the strong electron withdrawing nitro group has also been associated with increased protonophore activity which is believed to be the underlying mechanism by which niclosamide affects ATP synthesis and oxidative phosphorylation [44]. Therefore, we hypothesized that by removing the nitro group we may disrupt the mitochondrial uncoupling activity of niclosamide. We also aimed to improve potency and investigated the balance between the polar, hydrophilic carboxylic acid group and the non-polar lipophilic substituent at the 5-position of the salicyclic acid portion of the molecule by replacing the chloro group with alkyl groups.

**Fig 2:**
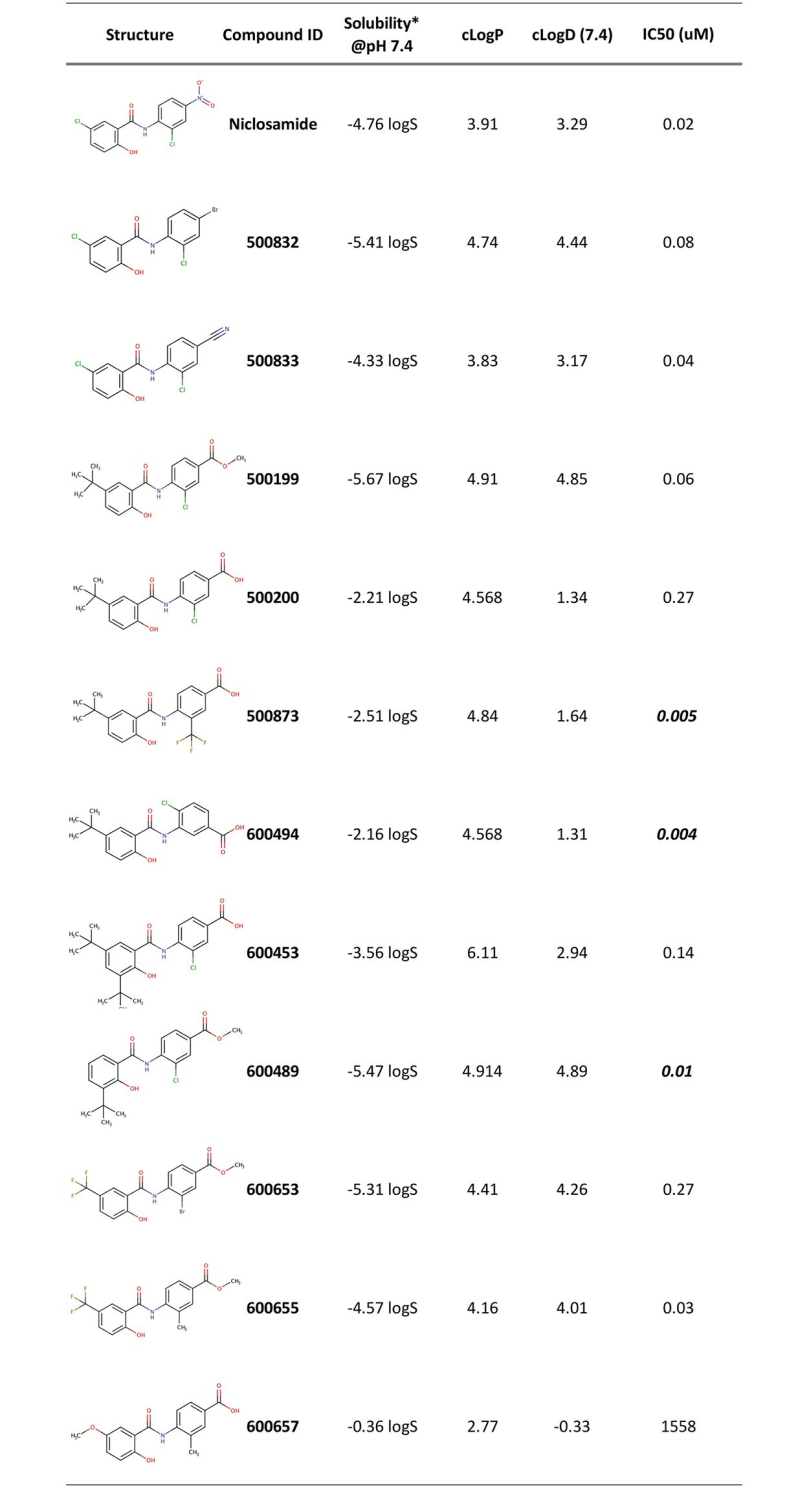
Structure and activity of N-substituted phenylbenzamides in DNL assay. The predicted aqueous solubility* at pH 7.4, cLogP, and cLogD (pH 7.4) were calculated using Instant Jchem or MarvinSketch. Inhibition of steatotic phenotype was quantified in PH5CH8 liver cells using the DNL assay. Lipid synthesis was stimulated in triplicate, in the presence of compounds in 10-point dose response between 1μM and 1.9nM. Cells were fixed, labeled and analyzed by high content image analysis. Partial least squares (PLS) regression analysis was used to determine % effect relative to DMSO controls. IC_50_ values were calculated for each compound.

Using this SAR strategy, we synthesized and tested 195 N-substituted phenylbenzamide derivatives in the DNL assay (S1 Table). All newly synthesized compounds were tested in dose response format using 10-point, 2-fold dilutions ranging between 1μM and 1.9nM. Compounds were considered active if they exhibited >45% effect in the DNL assay and if they displayed a sigmoidal dose response. Of those tested, 52 compounds were designated as active. Fig 2 shows data from 11 representatives of the N-substituted phenylbenzamide class. The predicted solubility at pH 7.4, clogP and cLogD values and IC_50_ for each derivative are shown. We compared the IC_50_ of each of the 11 compounds with that of niclosamide. 500873, 600494 and 600489 were found to be significantly more potent than niclosamide, with IC_50_ values of 0.005, 0.004 and 0.01μM respectively. In contrast, 600657 was by far the least potent compound with an IC_50_ of 1.6mM. Of note, several of the compounds including 500200 and 500873 produced inverse concentration curves. Of the 11 compounds, 7 had greater predicted solubility compared to niclosamide that was -4.76 logS. In particular, the predicted solubility of 600657 was best with a value of -0.36 logS which was followed by 600494 (-2.16 logS), 500200 (-2.21 logS) and 500873 (-2.51 logS) (Fig 2). Calculated cLogP values, a measure of hydrophobicity, are also shown in Fig 2 and with the exception of 600657 all compounds were more lipophilic compared to niclosamide and all of them are below 5.0 with the exception of 600453 with a clogP value of 6.1.

### Increase in cellular oxygen consumption rate (OCR) in intact cells

**Fig 3:**
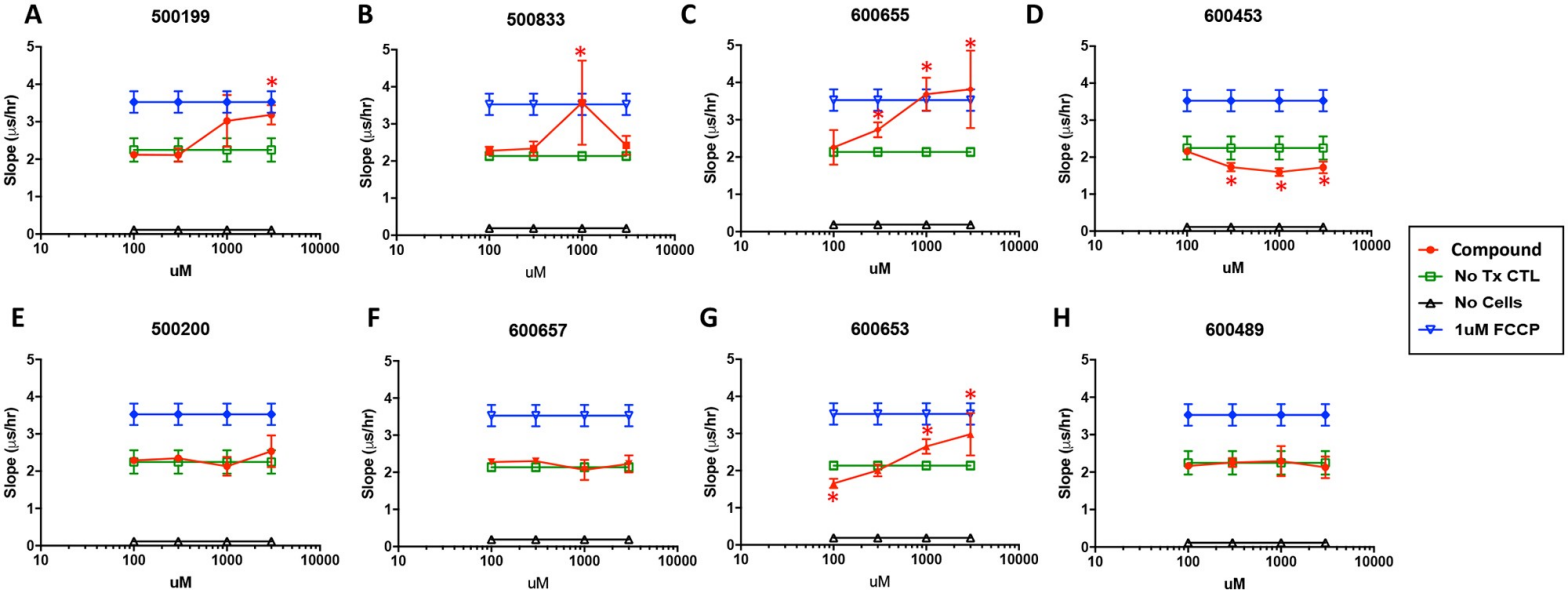
Dose dependent changes in oxygen consumption rate (OCR). Cellular respiration rate was measured in HEK293 cells expressing human OCT1 using the Mitoexpress-Xtra kit. The lifetime slope was plotted against concentration of compound. Oxygen consumption measurements were obtained immediately after treatment and for a period of 2 hours. The slope was calculated from the linear portion of the 2-hour kinetic run. Basal respiration (no treatment, green), stimulated respiration (1uM FCCP, blue) and no cell control (black) are shown. Changes with * indicate statistically significant (p<0.05) OCR values compared to basal OCR values.

Both niclosamide and its salt forms uncouple mitochondrial oxidative phosphorylation and this is characterized by an induction of cellular Oxygen Consumption Rate (OCR) [3]. Having identified a number of phenylbenzamides with *in vitro* activity for attenuating the hepatic steatosis phenotype, we next explored the effect of these structural changes on OCR using the MitoXpress Xtra-Oxygen Consumption Assay kit (Luxcel Biosciences). Due to the very low basal respiration rate of PH5CH8 cells, OCR was measured in a HEK293 cell line stably expressing human OCT3. As positive controls, we included carbonyl cyanide-p-trifluoromethoxyphenylhydrazone (FCCP), a well-known uncoupler of oxidative phosphorylation, and niclosamide, both of which increased OCR in cells compared to the DMSO control were tested alongside select phenylbenzamide derivatives (Fig 3). Interestingly, the phenylbenzamides derivatives produced varied responses in the OCR assay ranging from no change in respiration for 500200, 600657, 600489, 500873, and 600494 to significant changes for 500199, 600655, 600653, 500833, and 500832. Niclosamide, 600655, 600653, and 500199 showed a dose dependent increase in OCR whereas 500833 and 500832 were only significantly elevated at 1μM. In contrast to other phenylbenzamide derivatives, 600453 significantly decreased OCR at all concentrations above 300nM.

### Activation of AMP-activated protein kinase

**Fig 4:**
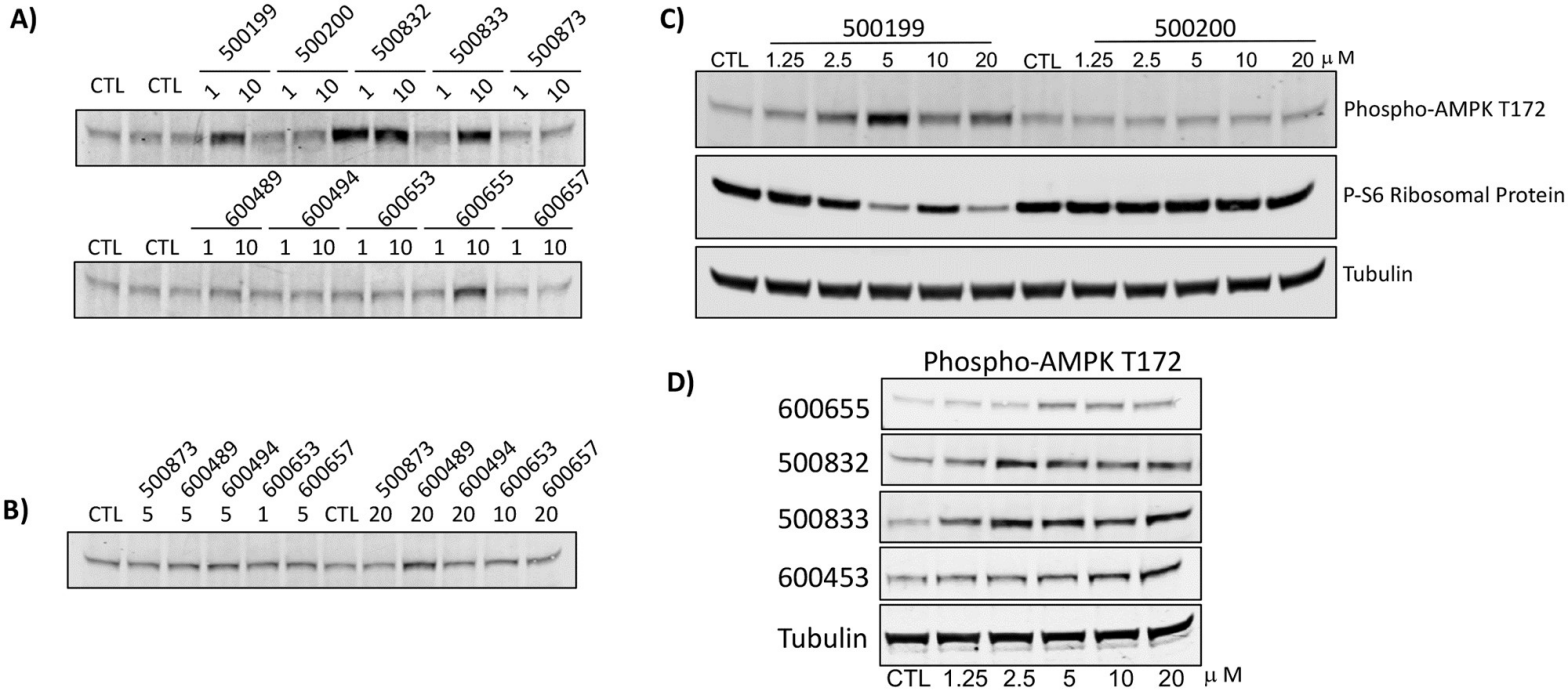
Validation of AMPK pathway activation by phenylbenzamide derivatives in HepG2 cells. A) Cells were seeded at 10,000 cells per well 24 hours prior to treating cells with indicated concentration of compound or DMSO (CTL) for 4 hours. 16μL of cell lysate was incubated with HTRF phospho-AMPK detection reagents and fluorescence emission read at 665nm and 620nm using a PHERAstar platereader. The mean and standard deviation of the HTRF Ratio from three replicate wells is shown. The unstimulated (DMSO) and positive controls lysates are shown in green and red respectively.

**Fig 5:**
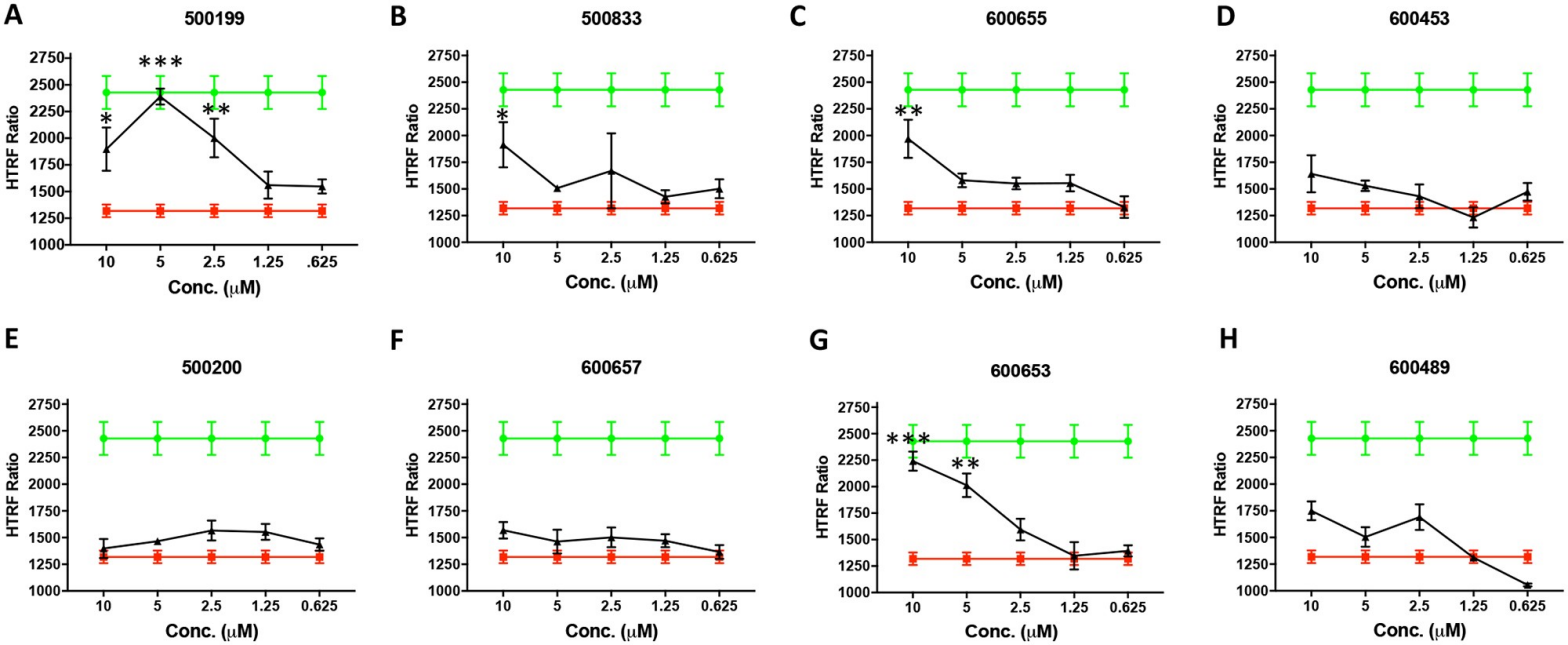
Validation of AMPK pathway activation by phenylbenzamide derivatives in HepG2 cells. A) Cells were seeded at 10,000 cells per well 24 hours prior to treating cells with indicated concentration of compound or DMSO (CTL) for 4 hours. 16μL of cell lysate was incubated with HTRF phospho-AMPK detection reagents and fluorescence emission read at 665nm and 620nm using a PHERAstar platereader. The mean and standard deviation of the HTRF Ratio from three replicate wells is shown. The unstimulated (DMSO) and positive controls lysates are shown in green and red respectively.

AMPK is a metabolic sensor that maintains cellular energy supply. As validation of these changes in lipid metabolism and cellular respiration we evaluated phospho-AMPK Thr-172 (p-AMPK) levels by Western blot in HEK293 cells treated with the phenylbenzamide derivatives. Compounds were screened at 1 and 10μM using the known uncouplers niclosamide and FCCP as positive controls (Fig 4A). Compounds that showed no activation were re-screened at 5 and 20 μM to confirm that they were negative (Fig 4B). Compounds that stimulated phosphorylation of AMPK were evaluated in HepG2 cells using CisBio’s pAMPK Thr-172 TR-FRET assay in dose response between 0.625 and 10 μM (Fig 5). Western blot analysis of p-AMPK Thr-172 in HEK293T cells showed that 500199 but not its acid form 500200 stimulated phosphorylation of AMPK peaking at 5 μM and corresponding phospho-S6 ribosomal protein was inversely regulated (Figs 5 and 4C). 600655, 500832, 500833, 600653 and 600453 also stimulated phosphorylation of AMPK albeit with different potencies (Figs 5 and 4D). Compounds 500873, 600657 showed no detectable changes in pAMPK levels. We concluded that compounds that had little effect on oxygen consumption rates also had little effect on phosphorylation of AMPK. However, 600453 appeared to stimulate phosphorylation of AMPK without changes in oxygen consumption. Compound 600657 did not appear to stimulate phosphorylation of AMPK or increase oxygen consumption rate and 600489 stimulated phosphorylation of AMPK only at the highest concentration but had no effect on oxygen consumption rate at the concentrations tested.

### Microsomal stability and pharmacokinetic profiles

Metabolic stability is paramount to developing an oral therapeutic for NAFLD/NASH. We tested the *in vitro* intrinsic clearance of select compounds in human liver microsomes (HLM) and mouse liver microsomes (MLM) using verapamil as a control for poor stability (Table 1). Verapamil performed as expected as assessed by the percentage of the initial concentration of compound remaining after 1 hour with only 7.5% and 4.5% remaining after 60 min in HLM and MLM preparations respectively. In general, all other compounds exhibited good microsomal stability. As anticipated, compounds were more stable in MLM relative to HLM. 600494 exhibited the greatest metabolic stability with 99.4% and 109.6% of the initial concentration remaining after 60 minutes in HLM and MLM respectively. Similarly, 84.5% and 101.2% of the starting material remained after 60 minutes in HLM and MLM respectively for 500200. On the other hand, 600453 and 500873 were stable in MLM, but in HLM only 58.8% and 64.2% of the initial concentration remained after 60 minutes (Table 1). Of the compounds reported here, only 600489 was found to be completely degraded after 60 min with 3.1% remaining in HLM and 2.7% remaining in MLM.

**Table 1:** Metabolic stability of phenylbenzamides in human and male mouse liver microsomes.

Compound ID	Species	Assay Format	Remaining Percentage of 60 min (%)
Verapamil	Human	Tested Sample	7.5
		Negative Control	96.9
	Mouse	Tested Sample	4.5
		Negative Control	99.6
600453	Human	Tested Sample	58.8
		Negative Control	92.2
		Heat-inactivated Control	93.7
	Mouse	Tested Sample	88.1
		Negative Control	109.7
		Heat-inactivated Control	91.4
600489	Human	Tested Sample	3.1
		Negative Control	3.8
		Heat-inactivated Control	66.6
	Mouse	Tested Sample	2.7
		Negative Control	4.1
		Heat-inactivated Control	57.6
500200	Human	Tested Sample	84.5
		Negative Control	110.2
		Heat-inactivated Control	96.6
	Mouse	Tested Sample	101.2
		Negative Control	111.5
		Heat-inactivated Control	102.7
500873	Human	Tested Sample	64.2
		Negative Control	97.7
		Heat-inactivated Control	91.1
	Mouse	Tested Sample	107.5
		Negative Control	106.4
		Heat-inactivated Control	105.5
600494	Human	Tested Sample	99.4
		Negative Control	92.8
		Heat-inactivated Control	114.2
	Mouse	Tested Sample	109.6
		Negative Control	68.9
		Heat-inactivated Control	98.5

We next explored pharmacokinetics profiles in Sprague Dawley rats following oral and IV administration of four of these compounds that had previously exhibited good microsomal stability. In a previous study, a 5mg/kg oral dose of niclosamide in rats leads to a peak plasma concentration (Cmax) of 354ng/ml and half-life of 6.7 hours [45]. Three of the four phenylbenzamide derivatives had significantly improved oral bioavailability compared to niclosamide. Indeed, a single oral dose of 5mg/kg of 600453 formulated in PBS gave a peak plasma concentration of 2050ng/ml in rats with the half-life of 3.9 hours which correlated to 64%F. Similarly, oral administration of 600494 led to a peak plasma concentration of 1850ng/ml and 5.3-hour half-life and 14%F while, 500873 reached a peak of 912ng/ml and 3.3-hour half-life resulting in 10%F (Table 2). Therefore, by selectively modifying the phenylbenzamide functional groups, we were able to significantly improve the bioavailability by up to 30 times that of niclosamide. The half-lives (t_1∕2_) of 600453 and 600494 in rats were 3.9 and 4.6 hours respectively and greater than 1.5 hours more than that reported for NEN in mice[3, 46]. These values remain high enough to be suitable for advancement to clinical development.

**Table 2:** Pharmacokinetic parameters for phenylbenzamides after 1 mg/kg intravenous injection or 5mg/kg oral dose in Sprague Dawley rats.

**IV Pharmacokinetic Parameters**					
**PK parameters**	**Unit**	Mean + SD (n = 3)			
		**600453**	**500200**	**500873**	**600494**
Cl_obs	mL/min/kg	3.11 +/- 0.42	8.63 +/- 0.78	1.33 +/- 0.31	0.91 +/- 0.068
T 1/2	h	3.68 +/- 0.13	1.66 +/- 0.48	2.87 +/- 0.86	5.36 +/- 2.00
C 0	ng/mL	2815.52 +/- 7.02	13770.28 +/- 2611	24104.18 +/- 3998	22902.67 +/- 4297
AUC last	h*ng/mL	5396.90 +/- 683	1938.47 +/- 166	12962.09 +/- 3068	18150.18 +/-1189
AUC Inf	h*ng/mL	5424.10 +/- 689	1940.10 +/- 166	12992.94 +/- 3068	18385.36 +/- 1400
AUC _%Extrap_obs	%	0.50 +/-0.114	0.13 +/- 0.016	0.249 +/- 0.151	1.23 +/- 1.05
MRT Inf_obs	h	3.22 +/- 0.32	0.26 +/- 0.048	1.42 +/- 0.29	2.39 +/- 0.69
AUCl ast/D	h*mg/mL	5396.90 +/- 683	1938.47 +/- 166	12962.09 +/- 3068	18150.18 +/- 1189
V ss_obs	L/kg	0.60 +/- 0.076	0.13 +/- 0.020	0.11 +/- 0.004	0.13 +/- 0.035
**PO Pharmacokinetic Parameters**					
**PK parameters**	**Unit**	Mean + SD (n = 3)			
		**600453**	**500200**	**500873**	**600494**
Dose	mg/kg	5	5	5	5
T 1/2	h	3.91 +/- 0.75	1.51 +/- 0.40	7.75 +/- 4.6	4.58 +/- 2.61
T max	h	4 +/- 0.0	3.33 +/- 1.15	3.33 +/- 1.15	5.33 +/-2.31
C max	ng/mL	2050 +/- 558	50.8 +/- 35.7	912.33 +/- 856	1850 +/- 674
AUC last	h*ng/mL	16940.77 +/- 2753	216.45 +/- 127	6425.63 +/- 4085	12440.38 +/- 2710
AUC Inf	h*ng/mL	17332.66 +/- 2846	219.73 +/- 1253	6947.67 +/-3685	13022.46 +/- 3519
AUC _%Extrap_obs	%	2.22 +/- 1.36	1.94 +/- 1.27	11.14 +/- 11.6	3.64 +/-4.73
MRT Inf_obs	h	7.40 +/- 0.44	4.04 +/- 0.68	10.02 +/- 5.6	7.89 +/- 1.49
AUC last / D	h*mg/mL	3388.15 +/- 551	43.29 +/- 25.3	1285.13 +/- 817	2488.08 +/- 542
F	%	63.91 +/- 10.5	2.26 +/- 1.29	10.20 +/-6.3	14.17 +/- 3.8

### Genotoxicity

Results from several studies indicate that niclosamide may damage DNA [9, 47, 48]. Herein we used the Ames assay to assess the ability of four phenylbenzamide analogs to induce DNA damage in bacteria. Niclosamide induced ≥ 2-fold increase in the mean number of revertant colonies at 1.5, 4 and 10 μg/well in the presence of S9 mix in strain TA98 (Table 3). The mean values of revertant colonies were 52.0, 108.3 and 194.7 and exceeded the negative control range. In contrast, niclosamide did not induce ≥ 2-fold increases for TA100, WP2 uvrA or ≥3-fold increases for TA1535 and TA1537 at any concentration tested (Table 3). Niclosamide also exhibited significant cytotoxicity in WP2 uvrA (pKM101) ≥400 μg /well both in the presence and absence of S9 mix as well as in strains TA98, TA100, TA1535 and TA1537 at concentrations ≥10 μg /well in the presence of S9 mix and ≥1.5 μg /well in the absence of the S9 mix.

**Table 3:** Mutagenicity of niclosamide and N-substituted phenylbenzamides using the Ames assay. The values shown represent the mean number of revertant colonies in the presence and absence of exogenous metabolic activation (Aroclor 1254 induced rat liver S9) from 3 replicate wells. The positive control chemicals were 2-Aminoanthracene (2-AA) for all S9 activation experiments, 2-nitrofluorene (2-NF 2) for TA98, SA for TA100 and TA1535, ICR-191 for TA-1537 and MNNG for WP2 uvrA. * indicates compounds that exhibit statistically significant increases in mean revertant colonies (p<0.05) as indicated by the Ames assay.

Compound	Concentration (ug/Well)	Mean Revertant Colony Counts									
		TA98		TA-100		TA-1535		TA-1537		WP2 uvrA (pKM101)	
		S9-	SP9+	S9-	SP9+	S9-	SP9+	S9-	SP9+	S9-	SP9+
**Niclosamide**	1.5	1.5	52.0	9.3	19.3	-	3.3	-	1.7	17.3	36.7
	4	-	108.3	-	36.7	-	3.7	-	2.0	20.7	44.0
	10	-	-	-	23.3	-	-	-	0.7	19.3	34.0
	25	-	-	-	2.3	-	-	-	-	14.7	31.7
	64	-	-	-	-	-	-	-	-	22.7	27.0
	160	-	-	-	-	-	-	-	-	17.7	24.7
	400	-	-	-	-	-	-	-	-	17.7	14.7
	1000	-	-	-	-	-	-	-	-	14.0	14.7
**Controls**	**Solvent**	**4.3**	**8.0**	**31.0**	**18.8**	**3.5**	**2.8**	**1.5**	**2.2**	**22.7**	**33.3**
	**Positive Control**	**210.0**	**290.7**	**133.7**	**93.7**	**103.3**	**24.0**	**72.3**	**11.0**	**328.0**	**345.3**
**600453**	1.5	4.3	5.0	35.0	17.3	4.0	2.7	0.7	2.3	31.7	36.0
	4	3.7	8.3	30.0	21.3	3.7	3.7	1.3	3.3	20.7	35.0
	10	3.3	7.7	33.3	27.0	1.7	2.7	2.3	1.7	29.3	31.7
	25	3.0	6.3	24.0	34.0	2.7	2.3	1.3	1.7	15.7	33.3
	64	6.0	8.7	26.3	31.7	1.7	3.0	0.3	1.0	23.0	28.0
	160	3.7	6.3	21.3	24.3	4.0	3.3	0.0	3.3	20.0	23.0
	400	2.0	7.3	8.0	29.3	2.7	2.0	1.0	0.7	9.0	15.3
	1000	3.7	4.0	2.7	15.7	2.7	2.0	0.3	3.7	3.7	9.0
**Controls**	**Solvent**	**5.0**	**7.5**	**34.0**	**22.7**	**3.5**	**2.7**	**2.8**	**1.5**	**26.8**	**35.0**
	**Positive Control**	**210.0**	**290.7**	**133.7**	**93.7**	**103.3**	**24.0**	**73.0**	**11.0**	**328.0**	**345.3**
**500200**	1.5	9.0	7.7	32.3	18.7	-	-	-	-	-	-
	4	12.0	6.3	25.7	19.7	-	-	-	-	-	-
	10	8.7	9.0	26.7	22.0	-	-	-	-	-	-
	25	9.0	7.3	28.0	22.0	-	-	-	-	-	-
	64	7.7	9.0	26.3	20.3	-	-	-	-	-	-
	160	5.0	11.0	24.0	27.7	-	-	-	-	-	-
	400	9.7	7.0	29.7	18.3	-	-	-	-	-	-
	1000	4.3	4.3	16.3	8.0	-	-	-	-	-	-
**Controls**	**Solvent**	**6.7**	**8.2**	**32.5**	**20.5**						
	**Positive Control**	**258.7**	**640.0**	**120.0**	**69.7**						
**600494**	1.5	7.3	8.7	30.0	20.7	-	-	-	-	-	-
	4	7.7	9.3	27.0	17.3	-	-	-	-	-	-
	10	7.3	7.7	25.0	18.0	-	-	-	-	-	-
	25	6.7	12.0	24.0	22.0	-	-	-	-	-	-
	64	6.0	8.0	33.0	16.3	-	-	-	-	-	-
	160	11.7	15.7	25.7	21.0	-	-	-	-	-	-
	400	14.0	10.3	22.3	21.3	-	-	-	-	-	-
	1000	10.7	11.3	17.7	20.3	-	-	-	-	-	-
**Controls**	**Solvent**	**6.7**	**10.0**	**29.0**	**18.5**						
	**Positive Control**	**258.7**	**640.0**	**120.0**	**69.7**						
**500873**	1.5	5.0	5.7	33.0	22.3	-	-	-	-	-	-
	4	9.0	6.0	31.7	18.3	-	-	-	-	-	-
	10	4.0	6.3	28.3	20.7	-	-	-	-	-	-
	25	7.7	9.3	25.0	16.7	-	-	-	-	-	-
	64	11.7	8.3	28.7	18.3	-	-	-	-	-	-
	160	10.0	10.3	25.0	22.7	-	-	-	-	-	-
	400	11.7	14.0	21.0	17.7	-	-	-	-	-	-
	1000	14.0	16.3	-	-	-	-	-	-	-	-
**Controls**	**Solvent**	**6.7**	**10.5**	**27.8**	**21.2**						
	**Positive Control**	**258.7**	**640.0**	**120.0**	**69.7**						

In contrast 600453 did not induce ≥ 2-fold increases (for TA98, TA100 and WP2 uvrA (pKM101)) or 3-fold (for TA1535 and TA1537) increases at any dose levels tested in the mean number of revertant colonies and no cytotoxicity was exhibited at any concentration relative to the negative control (Table 3). Similarly, 500200 did not exhibit ≥ 2-fold increases in the mean number of revertant colonies in strains TA98 and TA100 at any concentration tested when compared to the negative control (Table 3). However, cytotoxicity was exhibited at the highest concentrations in both strains in the presence and absence of the S9 mix. In contrast, while 500873 and 600494 did not induce ≥ 2-fold increases in revertant colonies in strain TA100, both analogs induced ≥2-fold increases in revertant colonies at concentrations of 400 μg/well for 600494 and 1000 μg/well for 500873 in strain TA98 in the absence of S9 mix. From these studies, we concluded that niclosamide was positive in the Ames assay whereas 600453 and 500200 were negative. Data from 600494 and 500873 were ambiguous.

### Niclosamide improves glucose tolerance and alters hepatic biomarkers in HFD-fed mice

**Fig 6:**
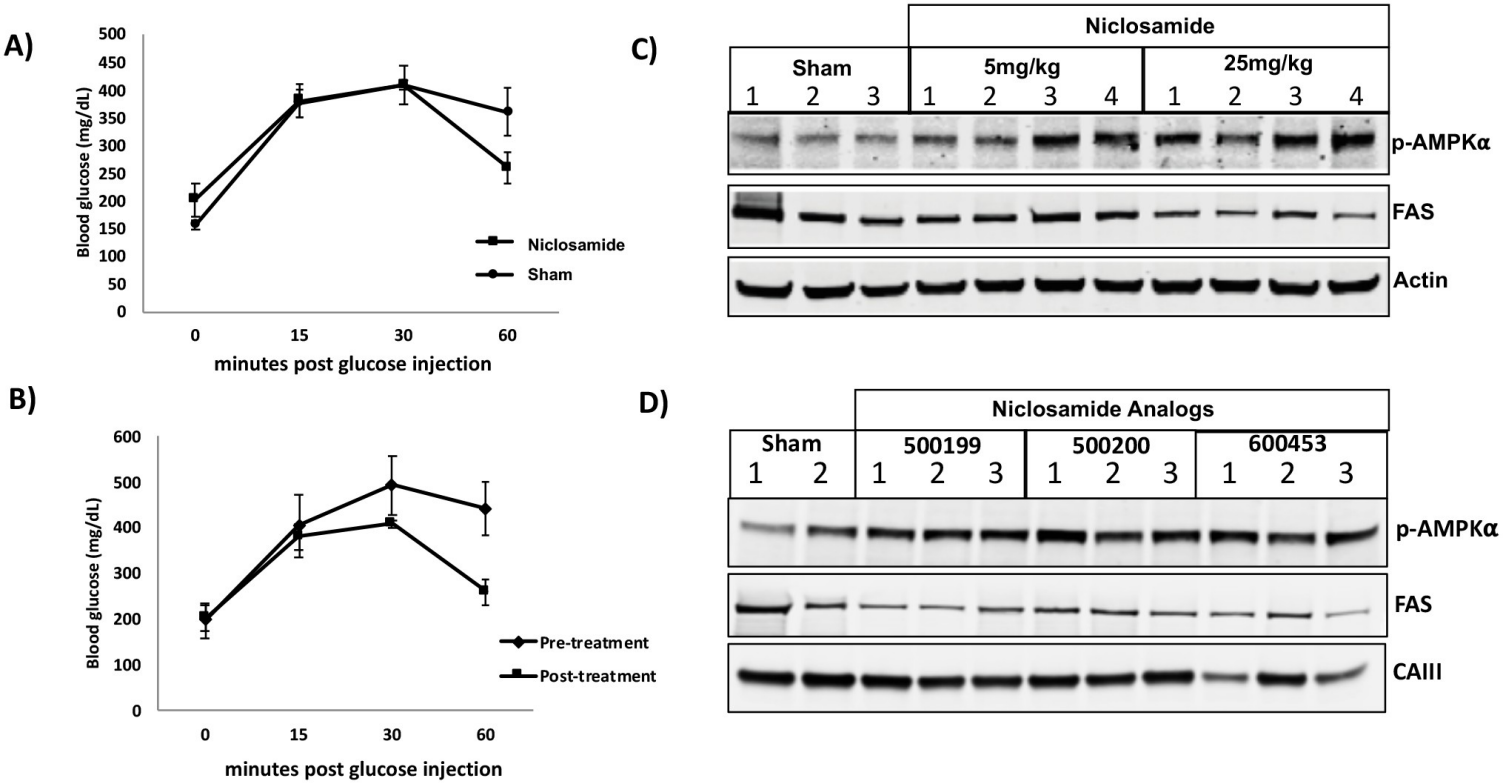
Niclosamide improves glucose tolerance and alters biomarkers of NAFLD and hepatic steatosis. Glucose tolerance test in DIO mice. 2g/kg of glucose was injected IP and at 15, 30 and 60 min, blood glucose levels were measured. **(A)** Averaged glucose tolerance for 7-day sham cohort and 5 mg/Kg Niclosamide treatment group (n = 3 each, P = 0.05). **(B)** Comparison of pre and post-7 day treatment with 5 mg/kg Niclosamide (n = 3, P = 0.034). **C)** Western blot analyses of phosphorylated AMPKα T172, Fatty acid synthase (FAS) and carbonic anhydrase III (CAIII) protein expression in liver tissue samples from male C57BL/6J mice fed HFD treated with 10mg/kg Niclosamide or D) 500200, 500199 or 600453 for 12 days relative to Sham HFD alone (control).

To compare the therapeutic effect of niclosamide and the newly synthesized analogs on hepatic steatosis, we used the C57-diet induced obesity (DIO) mouse as a disease model for both type 2 diabetes and extensive fatty liver disease. These mice exhibit massive hyperglycemia, obesity, hepatic steatosis, insulin resistance, dyslipidemia and pro-inflammatory cytokines by 16-20-weeks [49, 50]. Singly housed 20-week old male mice raised on a 60%-fat diet were tested for glucose sensitivity using the glucose tolerance test (GTT). Animals were then treated with 5mg/kg niclosamide or vehicle for 7 days and GTT was performed again. Compared to sham animals that had blood glucose levels of 350mg/dL at 60 minutes niclosamide treated animals exhibited statistically significant reduction in glucose levels to 250mg/dL (Fig 6A). Overall niclosamide reduced plasma glucose levels from approximately 400mg/dL to 275mg/dL over the 7-day period (Fig 6B). This is consistent with previous studies showing improved glucose metabolism in mouse models of metabolic disease [3, 24, 25].

We next measured body weight changes in HFD-fed animals prior to treatment with niclosamide or phenylbenzamide derivatives and after 12 days, prior to sacrifice. During this time, all animals (including sham) lost some weight. However, 500199 and 600453 treated animals lost significantly more weight when compared to sham animals in the same trial (Table 4). 500873 treated animals also lost weight but this loss failed to reach significance (p>0.05). No statistically significant differences were observed between sham and any other treatment groups.

**Table 4:** Niclosamide analogs attenuate hepatic steatosis in high fat diet fed mice. 20-week old HFD-fed mice were delivered vehicle or compound daily at 10mg/kg by I.P for 12 days. Body weight measurements and scoring of greasy coat phenotype were performed prior to and following treatment. Histological analysis of H&E stained liver sections were analyzed and the % of steatosis and steatosis score are reported.

Compound	Concentration (ug/Well)	Mean Revertant Colony Counts									
		TA98		TA-100		TA-1535		TA-1537		WP2 uvrA (pKM101)	
		S9-	SP9+	S9-	SP9+	S9-	SP9+	S9-	SP9+	S9-	SP9+
**Niclosamide**	1.5	1.5	52.0	9.3	19.3	-	3.3	-	1.7	17.3	36.7
	4	-	108.3	-	36.7	-	3.7	-	2.0	20.7	44.0
	10	-	-	-	23.3	-	-	-	0.7	19.3	34.0
	25	-	-	-	2.3	-	-	-	-	14.7	31.7
	64	-	-	-	-	-	-	-	-	22.7	27.0
	160	-	-	-	-	-	-	-	-	17.7	24.7
	400	-	-	-	-	-	-	-	-	17.7	14.7
	1000	-	-	-	-	-	-	-	-	14.0	14.7
**Controls**	**Solvent**	**4.3**	**8.0**	**31.0**	**18.8**	**3.5**	**2.8**	**1.5**	**2.2**	**22.7**	**33.3**
	**Positive Control**	**210.0**	**290.7**	**133.7**	**93.7**	**103.3**	**24.0**	**72.3**	**11.0**	**328.0**	**345.3**
**600453**	1.5	4.3	5.0	35.0	17.3	4.0	2.7	0.7	2.3	31.7	36.0
	4	3.7	8.3	30.0	21.3	3.7	3.7	1.3	3.3	20.7	35.0
	10	3.3	7.7	33.3	27.0	1.7	2.7	2.3	1.7	29.3	31.7
	25	3.0	6.3	24.0	34.0	2.7	2.3	1.3	1.7	15.7	33.3
	64	6.0	8.7	26.3	31.7	1.7	3.0	0.3	1.0	23.0	28.0
	160	3.7	6.3	21.3	24.3	4.0	3.3	0.0	3.3	20.0	23.0
	400	2.0	7.3	8.0	29.3	2.7	2.0	1.0	0.7	9.0	15.3
	1000	3.7	4.0	2.7	15.7	2.7	2.0	0.3	3.7	3.7	9.0
**Controls**	**Solvent**	**5.0**	**7.5**	**34.0**	**22.7**	**3.5**	**2.7**	**2.8**	**1.5**	**26.8**	**35.0**
	**Positive Control**	**210.0**	**290.7**	**133.7**	**93.7**	**103.3**	**24.0**	**73.0**	**11.0**	**328.0**	**345.3**
**500200**	1.5	9.0	7.7	32.3	18.7	-	-	-	-	-	-
	4	12.0	6.3	25.7	19.7	-	-	-	-	-	-
	10	8.7	9.0	26.7	22.0	-	-	-	-	-	-
	25	9.0	7.3	28.0	22.0	-	-	-	-	-	-
	64	7.7	9.0	26.3	20.3	-	-	-	-	-	-
	160	5.0	11.0	24.0	27.7	-	-	-	-	-	-
	400	9.7	7.0	29.7	18.3	-	-	-	-	-	-
	1000	4.3	4.3	16.3	8.0	-	-	-	-	-	-
**Controls**	**Solvent**	**6.7**	**8.2**	**32.5**	**20.5**						
	**Positive Control**	**258.7**	**640.0**	**120.0**	**69.7**						
**600494**	1.5	7.3	8.7	30.0	20.7	-	-	-	-	-	-
	4	7.7	9.3	27.0	17.3	-	-	-	-	-	-
	10	7.3	7.7	25.0	18.0	-	-	-	-	-	-
	25	6.7	12.0	24.0	22.0	-	-	-	-	-	-
	64	6.0	8.0	33.0	16.3	-	-	-	-	-	-
	160	11.7	15.7	25.7	21.0	-	-	-	-	-	-
	400	14.0	10.3	22.3	21.3	-	-	-	-	-	-
	1000	10.7	11.3	17.7	20.3	-	-	-	-	-	-
**Controls**	**Solvent**	**6.7**	**10.0**	**29.0**	**18.5**						
	**Positive Control**	**258.7**	**640.0**	**120.0**	**69.7**						
**500873**	1.5	5.0	5.7	33.0	22.3	-	-	-	-	-	-
	4	9.0	6.0	31.7	18.3	-	-	-	-	-	-
	10	4.0	6.3	28.3	20.7	-	-	-	-	-	-
	25	7.7	9.3	25.0	16.7	-	-	-	-	-	-
	64	11.7	8.3	28.7	18.3	-	-	-	-	-	-
	160	10.0	10.3	25.0	22.7	-	-	-	-	-	-
	400	11.7	14.0	21.0	17.7	-	-	-	-	-	-
	1000	14.0	16.3	-	-	-	-	-	-	-	-
**Controls**	**Solvent**	**6.7**	**10.5**	**27.8**	**21.2**						
	**Positive Control**	**258.7**	**640.0**	**120.0**	**69.7**						

In an attempt to understand the mechanistic basis for these changes we evaluated several pathways dysregulated in the livers of HFD-fed mice by Western blots analysis. Given the correlation between AMPK and lipid metabolism we explored the possibility that niclosamide activates AMPK *in vivo*. 20-week old male mice raised on a 60%-fat diet were treated with niclosamide at 5 or 25mg/kg for 12 days via a daily (IP) injection with DMSO as the vehicle. Sham animals were administered vehicle on the same schedule. To determine if AMPK is activated *in vivo* in response to niclosamide we measured p-AMPK Thr-172, as phosphorylation at this site is absolutely required for AMPK activity and widely used in the AMPK field as a surrogate for kinase activity [51, 52]. AMPK was significantly upregulated *in vivo* by low and high doses of niclosamide (Fig 6C). We also observed a significant reduction in the expression of fatty acid synthase (FAS) at the highest concentration of niclosamide (Fig 6C). It is of interest to note that in previous studies we also identified carbonic anhydrase-III (CAIII) as a hepatic protein altered in HFD fed-DIO mice that was down regulated by niclosamide treatment and positively correlates with the extent of ectopic lipid accumulation [31].

500199, 500200 and 600453 were also analyzed for effects on p-AMPK Thr-172, FAS and CAIII using Western blot analysis. These studies showed that treatment of HFD fed-DIO mice with 10mg/kg of 500199, 500200 and 600453 for 12 days also stimulated phosphorylation of AMPK at Thr-172, and reduced FAS and CAIII protein levels when compared to sham mice (Fig 6D).

**Fig 7:**
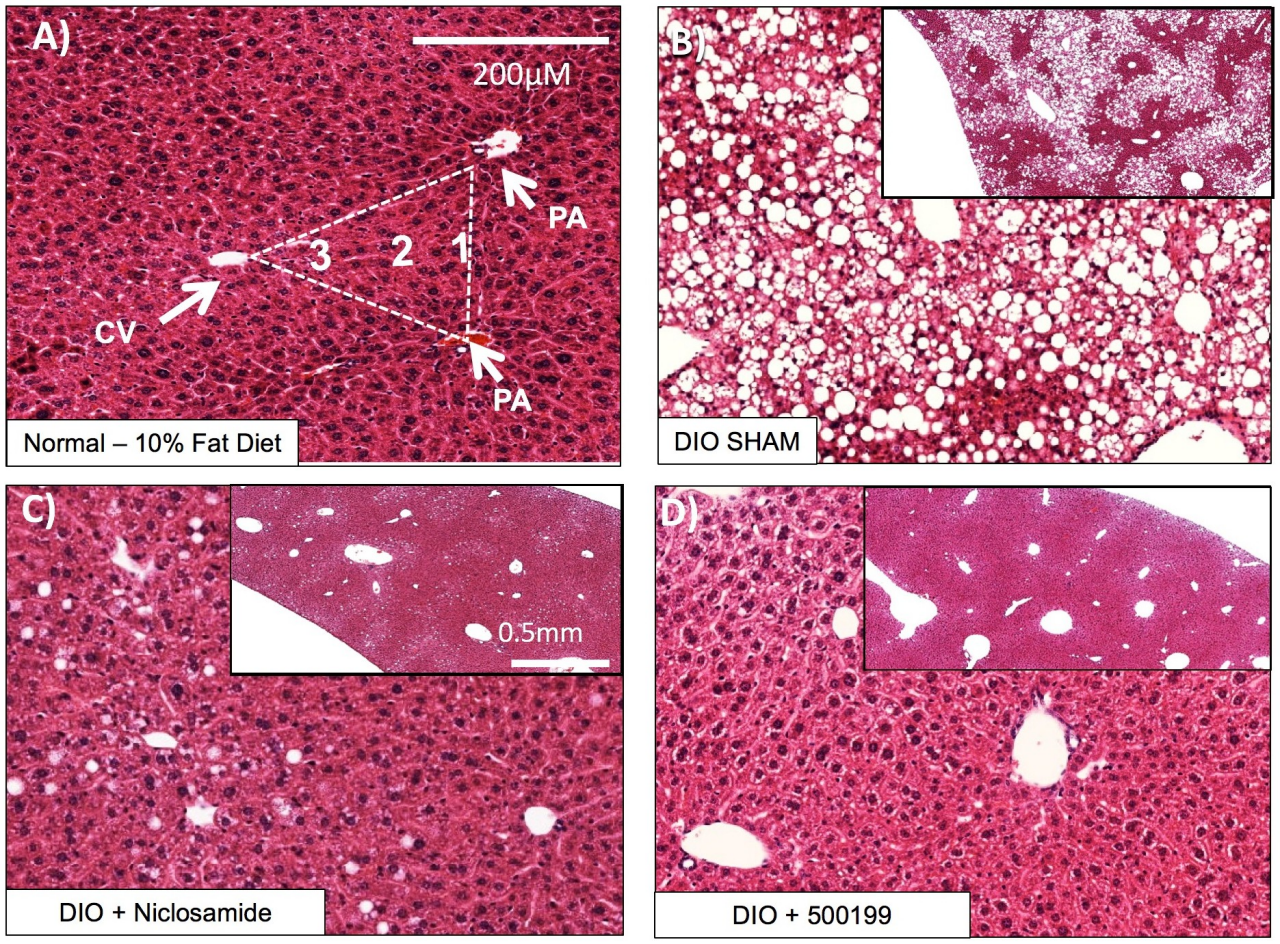
500199 attenuates hepatic steatosis in high fat diet fed mice. 20-week old HFD-fed mice were delivered vehicle or compound daily via intraperitoneal injection for 12 days and compared to sham mice fed 10% fat diet. Representative images of H&E stained liver sections from 10% fat diet mice (A) 60% HFD-fed mice (B) or 60% HFD-fed mice treated with niclosamide (C) or 500199 (D) at 10mg/kg.

Niclosamide and its salt forms NEN and NPP have been shown to improve hepatic steatosis in mouse models of metabolic disease [3, 24, 25]. To compare the ability of the novel benzamides to improve hepatic steatosis, 60%-fat fed DIO mice were treated with niclosamide or one if its derivatives for 12 days and the livers were immediately examined for changes in gross morphology. With the exception of liver from 500832 treated animals, all livers from treated animals showed improvements in morphology within 12 days. The livers of treated animals became darker red in color compared to the sham HFD-fed animals, which were visibly lighter and tan in color due to ectopic fat accumulation. Histological analysis of the lower left lobe from all treated mice showed an improvement in macro- and micro- steatosis within 12 days, with the exception of mice treated with 500832. The pattern of steatosis was also altered, with animals treated with niclosamide and 500199 being almost completely cleared of steatosis. Most notably, clearance of lipids was observed predominately from zone-1 in 500199 treated animals. Representative images of liver tissues from mice fed standard chow (10% fat diet) or HFD–fed animals (60% HFD) treated with niclosamide, 500199 or Sham animals are shown in Fig 7A–7D. The steatosis score was used to evaluate the livers, scoring microvesicular or macrovesicular steatosis and fatty infiltration zonality [32]. Niclosamide treated animals had variable degrees of steatosis ranging from 15–70% with a mean steatosis of 45% and a steatosis score of 2.0 (Table 4). In contrast, sham animals typically had greater than 70% steatosis resulting in a steatosis score of 3.0 (Table 4). Similar to niclosamide, 600453 and 500199 reduced the mean percentage of steatosis to 38% and 19% respectively resulting in a steatosis score of 1. Other analogs including 600489 (24% steatosis), 500200 (28% steatosis), 500873 (28% steatosis), and 600494 (25% steatosis) also exhibited reduced steatosis with a score of 1.25 (Table 4). In some cases, livers more closely resembled livers from standard chow fed mouse with ≤5% steatosis despite being maintained on the HFD during the 12 days of treatment. 600653 and 500832 had a more modest effect on hepatic steatosis compared to other phenylbenzamides tested and these had a steatosis score of 1.75 and 2.75 respectively.

**Fig 8:**
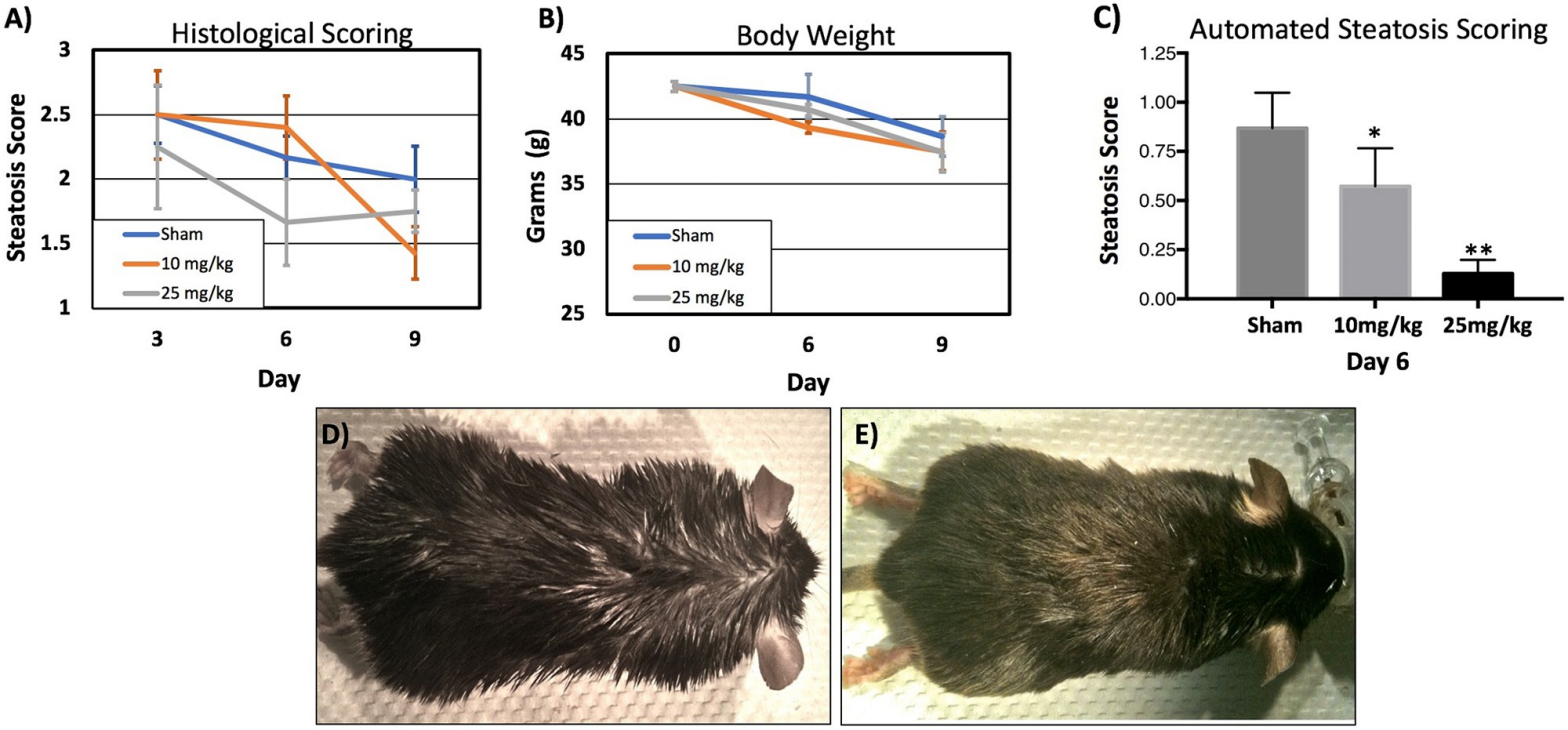
500199 attenuates hepatic steatosis and improves greasy coat phenotype in high fat diet fed mice but has no effect on body weight. Histological scoring of steatosis in H&E stained whole liver sections (A) and body weight in grams (B) of HFD-fed mice with and without 500199 administration at 10 or 25mg/kg by I.P for 9 days. Steatosis scoring using automated image analysis pipeline for the identification of macro- and micro- steatosis in H&E stained whole liver sections (C). Tabulated lipid droplet statistics for sham vs. 500199 treated liver tissues on Day 6. * indicates statistically significant differences p<0.02 ** indicates statistically significant differences p<0.0001. (E) Greasy coat phenotype in 20-week old HFD-fed mice (D) and clearing of greasy coat phenotype in HFD-fed mice treated with 500199 (E).

20-week old animals were treated IP with 500199 at 10 or 25mg/kg by IP over a time-course of 9 days. At day 0, 3, 6 and 9 animals were weighed and tissue samples were collected for standard histological scoring of hepatic steatosis and automated quantitative histomorphometric scoring. Using the standard histological scoring method, all 500199 treated animals showed a time and dose dependent improvement in steatosis score (Fig 8A). In addition, all animals showed a gradual decline in body weight. However no statistically significant difference was observed between treatment groups and sham animals (Fig 8B).

We then used an Aperio ScanScope slide scanner to scan H&E stained slides for image analysis and quantification of macrovesicular and microvesicular steatosis. We used this approach to compare the effect of low and high dose 500199 on hepatic steatosis phenotype on day 6 of treatment, where the most differences were observed in histological analysis. Animals treated with 500199 showed a dose dependent improvement in steatotic phenotype with a score of 0.57 ± 0.19 (p≤0.018) and 0.13 ± 0.17 (p≤0.0001) for 10 and 25mg/kg doses respectively. This quantitative analysis can be used along-side comprehensive manual histological scoring to measure changes in the steatotic phenotype including changes in macrosteatosis and microsteatosis, lipid droplet size, zonal lipid distribution, and lipid droplet count.

In addition to changes in hepatic steatosis, we regularly observed that the coats of treated animals were drier, consistent with reduction in triglycerides, as has been observed in the DGAT1 mouse knockout with dry coat (Fig 8D and 8E) [53]. Animals were scored with regards to their oily coat phenotype with +++ representing an oily coat and ++ and + progressively drier phenotype. Although the effect was difficult to quantify as the effect was greater in some animals than others, in general we observed changes in coat appearance with niclosamide, 500199 and 500832 treated animals (Table 4).

## Discussion

Herein we report the development of a cell-based high-throughput assay suitable for identifying compounds that improve a fatty liver phenotype. We used this platform to identify several novel phenylbenzamide derivatives of the niclosamide chemotype that improve hepatic steatosis *in vitro* and in HFD-fed obese mice with prominent fatty liver disease.

Previous studies have described the ability of niclosamide and its salt forms to treat the diabetic symptoms and fatty liver in mouse models of metabolic disease [3, 25]. While the exact mechanism underlying niclosamide’s therapeutic effect remains unclear, both studies appear to associate the anti-hyperglycemic effect with the ability of niclosamide and its salts to uncouple oxidative phosphorylation. This finding is supported by the observations that sulfonate conjugated niclosamide (SCN), a derivative of NEN containing a sulfonate conjugate at the 2-hydroxyl site essential for mitochondrial uncoupling, failed to improve hyperglycemia *in vivo*. This finding led the authors to conclude that mitochondrial uncoupling is required for anti-hyperglycemic activity [3]. Unfortunately, the study did not address the effect of SCN on hepatic steatosis and it remains to be determined whether SCN or other niclosamide derivatives that do not uncouple oxidative phosphorylation or stimulate cellular respiration can improve hepatic steatosis through an independent pathway. Consistent with this idea, Mook et. al., recently showed that inhibition of Wnt/B-catenin signaling induced by niclosamide analogs can be separated from uncoupling of oxidative phosphorylation and alterations in cellular ATP [54].

The uncoupling of oxidative phosphorylation leads to increased oxygen consumption without a concomitant rise in ATP production. Niclosamide and its salt forms uncouple mammalian mitochondria at high nanomolar concentrations and increase cellular OCR in cells without stimulating hyperthermia as with other mitochondrial uncouplers [3]. Mechanistically this may lead to increased energy expenditure and lipid oxidation as well as lower levels of cAMP that are required for PKA mediated hepatic glucagon signaling [3, 25]. Interestingly, differences in both energy expenditure and body weight were reported in response to niclosamide and its salts. Tao et. al., reported increased energy expenditure and reduction in body weight while Chowdhury et. al., observed no changes in body weight or energy expenditure but rather, attributed the anti-hyperglycemic effect to decreased hepatic glucose production as a result of the ability of niclosamide to block glucagon signaling in the liver. The requirement of uncoupling for clearing hepatic steatosis was not directly tested in these studies.

While improvement of diabetic outcomes was not a focus of this paper, in our hands niclosamide improved glucose tolerance and this is consistent with previous studies that show improved glucose tolerance in the absence of changes in energy expenditure with niclosamide treatment (Fig 5)[25, 55].

We synthesized and screened 197 phenylbenzamide derivatives of the niclosamide chemotype and tested their ability to attenuate hepatic steatosis *in vitro*. This approach identified a number of novel compounds that attenuate hepatic steatosis both *in vitro* and *in vivo* in the low nanomolar range but that fail to increase oxygen consumption at concentrations up to 3μM. While we did not directly test uncoupling of oxidative phosphorylation in isolated mitochondria, increased oxygen consumption has been observed with niclosamide, NEN and NPP at sub-micromolar concentrations [3, 24, 25]. This finding suggests that it may be possible with SAR elucidation to separate the effect on hepatic steatosis from any potential mitochondrial uncoupling activity and related effects on glucose metabolism [3]. Changes in body weight were observed with administration of some of the phenylbenzamide derivatives, however additional experiments will be necessary to draw any conclusions regarding mitochondrial uncoupling and energy expenditure.

Niclosamide is minimally absorbed from the gastrointestinal tract leading to low oral bioavailability. Consequently, there is a need to identify novel derivatives with improved bioavailability to treat conditions that may require systemic exposure. Such compounds could provide new options to treat a diverse set of indications, including NAFLD, diabetes and metabolic disease, viral infection and a variety of cancers. Herein we report the bioavailability of several compounds has been improved by achieving a balance between the polar, hydrophilic carboxylic acid group and the non-polar, lipophilic tert-butyl group as exemplified in 500200 and 600453. The half-life of niclosamide is 6.7 hours [56]. Several of the phenylbenzamides reported in this study show both improved oral bioavailability and a half-life of greater than 4 hours suggesting they could be amenable to a once daily oral delivery. Furthermore, a number of the compounds reported herein have greater cLogP values and significantly improved cLogD values as compared to niclosamide. For instance, the introduction of more polar surface area with the methoxy group in 600657 gives a cLogD of -0.33. Niclosamide and NEN accumulate predominately in the liver. Further studies will be needed to explore how these differences affect the tissue distribution and plasma protein binding of the compounds reported in this study. Niclosamide also has a potentially toxic aromatic nitro group and in all of the newly reported compounds, the group has been replaced with a carboxylic acid moiety with retention of activity in our assays.

Although niclosamide has a good safety profile in humans when given as a single oral dose, niclosamide leads to an increase in sperm head abnormalities, clastogenic effects on human lymphocytes, and frame shift mutations in salmonella sp. [47, 48, 57]. It is unclear if these effects on DNA will become problematic with chronic treatment strategies that would be required to treat fatty liver disease or diabetes. Herein we identified several compounds (600453, 600494 and 500873) that, in contrast to niclosamide, are negative in the Ames assay, a measure of mutagenicity. Together these factors of improved pharmacokinetic properties and retention of important biological activity, in the absence of elevated respiration and low potential genotoxicity, suggest that compared to niclosamide, they may be better therapeutic candidates for the treatment of NAFLD and diabetes.

Niclosamide has been tested against a panel of 95 protein kinases at concentrations that inhibit the Wnt/ß Catenin, mTORC, STAT3, NFκB and NOTCH signaling pathways but it did not significantly inhibit any of these kinases, suggesting that it is unlikely to be a direct inhibitor of any kinases [44]. We recently identified carbonic anhydrase 3 (CAIII) as one of three most influenced genes upregulated in mice in response to HFD and that niclosamide reversed CAIII upregulation [58]. Similarly, we noted that of the phenylbenzamides that we tested *in vivo* all three reduced the hepatic CAIII expression as well as FAS suggesting that CAIII may be a suitable biomarker for measuring the beneficial effects of potential therapeutics on hepatic steatosis *in vivo*.

Due to AMPK’s direct role in inhibiting fatty acid synthesis, AMPK has also been shown to be a promising target for treatment of both alcoholic and non-alcoholic fatty liver disease [59] [60]. It is a highly-conserved serine threonine kinase that regulates cellular metabolism. The most characterized AMPK downstream target, acetyl coenzyme A carboxylase (ACC), is a rate-limiting enzyme in energy consumptive *de novo* fatty acid synthesis, and ACC is directly inhibited by AMPK phosphorylation to conserve cellular energy. Niclosamide and NEN have both been shown to activate AMPK in a dose and time-dependent manner resulting in an increase in ACC phosphorylation and inhibition of mitochondrial beta oxidation. Therefore, it is possible that by activating AMPK the compounds described herein could increase lipid oxidation through downstream phosphorylation and inhibition of ACC and mitochondrial beta oxidation.

Consistent with this idea, we identified several phenylbenzamides (500199, 500832, 500833 and 600653) that were associated with increased respiration and increased phosphorylation of AMPK at threonine 172 (T172) and that attenuated hepatic steatosis in liver cells. This may suggest that these compounds act primarily through uncoupling mitochondria leading to activation of the AMPK pathway and to increased lipid oxidation, similar to niclosamide and its salt forms. In contrast, we also identified several compounds that attenuated hepatic steatosis (500200, 600657, and 600489) in the absence of changes in the oxygen consumption rate and activation of the AMPK pathway *in vitro*. It may be possible that these compounds work through a, as yet undetermined but distinct mechanism. Irrespective of the mechanism of action, all of these compounds showed efficacy in the HFD-fed obese mouse model and attenuated hepatic lipid accumulation.

## Conclusions

In conclusion, niclosamide presents a number of potential challenges to becoming a safe and effective drug for extended therapeutic use. In an effort to identify novel compounds with better preclinical properties that retain *in vivo* efficacy for the treatment of hepatic steatosis and NAFLD, we synthesized a series of novel N-substituted phenylbenzamide derivatives of the niclosamide chemotype. This study describes a series of novel phenylbenzamide derivatives with improved physicochemical and pharmacokinetic characteristics that attenuate hepatic steatosis *in vitro* and *in vivo* for the treatment of fatty liver and NAFLD. Furthermore, our data suggest that the compounds might be separated into two groups those that increased respiration and stimulated activation of the AMPK pathway and those that do not. Both groups were equally as effective in attenuating hepatic steatosis *in vitro* and *in vivo* suggesting the uncoupling of oxidative phosphorylation is not required for the treatment of hepatic steatosis. In particular, we identified 600453 as a N-substituted phenylbenzamide that has good oral bioavailability and half-life, lacks genotoxicity, and does not stimulate respiration in cells. This suggest that this compound may be a good candidate for further clinical development for fatty liver disease.

## Supporting information

S1 File Synthetic chemistry procedures. (DOCX)

S1 Table SAR for N-substituted phenylbenzamides. (XLSX)

S1 Fig (TIF)
