# Supplementary material for: N-substituted phenylbenzamides of the niclosamide chemotype attenuate obesity related changes in high fat diet fed mice
Source: PLoS One. 2018 Oct 25;13(10):e0204605. doi: 10.1371/journal.pone.0204605 (PMC6201879; doi:10.1371/journal.pone.0204605)
Supplement: S1 File — (DOCX) [file pone.0204605.s001.docx]

**Synthetic Chemistry Procedures:**

**500832: N-(4-Bromo-2-chlorophenyl)-5-chloro-2-hydroxybenzamide:**

A mixture of 5-chlorosalicylic acid (0.40 g, 2.3 mmol) and 4-bromo-2-chloroaniline (0.48 g, 2.3mmol) in Xylenes (10 mL) was heated to 122^o^C. PCl_3_ (0.46 mL, 0.62 mmol) as a 2M solution in dichloromethane was added dropwise via syringe. The mixture was heated at 130^o^C for 4 hr. The temperature was reduced to 100^o^C and the hot solution was pipetted into an Erlenmeyer flask and vigorously stirred while cooling to room temperature. The resulting solid was filtered, washed several times with hexanes and air dried to get 0.61 g of crude product as a white solid. The solid was dissolved in ethyl acetate, silica gel was added and the mixture was concentrated in vacuo. The residue was transferred to a pre-column and purified by flash chromatography using a gradient as follows: Hexanes to 30% Ethyl Acetate/hexanes over a 2 min period, then 30% Ethyl Acetate/hexanes for the remainder of the purification. The fractions containing the pure major component were combined and concentrated in vacuo to give 0.43 g (52%) of N-(4-bromo-2-chlorophenyl)-5-chloro-2-hydroxybenzamide as a white solid.

MS: m/z 357.9 (MH^-^) and m/z 359.9 (MH^+^).

^1^H NMR (500 MHz, DMSO-d_6_): δ 7.077 (d, 1H), 7.516 (dd, 1H), 7.609 (dd, 1H), 7.849 (d, 1H), 7.972 (d, 1H), 8.383 (d, 1H), 10.920 (s, 1H), 12.288 (s, 1H).

**500833: 5-Chloro-N-(2-chloro-4-cyanophenyl)-2-hydroxybenzamide:**

To a mixture of 5-chloro-2-hydroxybenzoic acid (0.35 g, 0.002 mol) and 4-amino-3-chlorobenzonitrile (0.31 g, 0.002 mol) in xylenes (10 mL) at ambient temperature was added phosphorous oxychloride (0.068 g, 0.56 mmol) in one portion. The mixture was refluxed for 17.5 h. The mixture was cooled to ambient temperature and the resulting orange solid was filtered and washed several times with hexanes to give 265 mg of crude product. The orange solid was placed in hot acetic acid (45 mL) and an insoluble orange solid (**B**) was filtered off. The filtrate was allowed to cool to ambient temperature and stand for 3 days. The resulting light orange solid (**A**) was filtered and washed with hexanes. After air drying both solids, **B** amounted to 33.8 mg and **A** amounted to 92.8 mg. Total yield is 126.6 mg (21%yield) of 5-Chloro-N-(2-chloro-4-cyanophenyl)-2-hydroxybenzamide. Both solids gave similar MS and NMR results. The following MS and NMR results are for **A**.

MS: m/z 305 (MH^-^).

^1^H NMR (500 MHz, DMSO-d_6_): δ 7.097 (d, 1H), 7.538 (dd, 1H), 7.884 (dd, 1H), 7.967 (d, 1H), 8.190 (d, 1H), 8.714 (d, 1H), 11.220 (s, 1H), 12.450 (s, 1H).

**500199**: **Methyl 4-[(5-tert-butyl-2-hydroxybenzene)amido]-3-chlorobenzoate:**

**Step 1: 2-Hydroxy-5-(tert-butyl)benzaldehyde:** The following procedure was adapted from that described in[1]: To a solution of 4-(tert-butyl)phenol (5.0 g, 0.033 mol) in acetonitrile (150 mL) at ambient temperature under N_2_ atmosphere was added triethylamine (12.1 g, 0.12 mol) followed by MgCl_2_ (4.8 g, 0.5 mol). The resulting mixture was stirred at ambient temperature for 15 min. Paraformaldehyde (6.6g, 0.22 mol) was added and the mixture was heated at reflux for 3h. The mixture was cooled to ambient temperature and poured into 5% aqueous HCl (260 mL). The mixture was stirred for 30 min. at ambient temperature then extracted several times with diethyl ether. The combined organic phase was washed with brine and dried over MgSO_4_.The mixture was filtered and concentrated in vacuo to give 5.7 g of a slightly cloudy yellow oil. The oil was purified by passing through a column of silica gel 2.5” tall and 3.5” in diameter (sintered glass funnel) using house vacuum to pull the solvent through. Initially hexanes (19 fractions ~100 mL each) were collected, then the eluent was modified to hexanes/diethyl ether (4:1) for the remainder of the purification. The fractions containing the pure fastest eluting main component were combined and concentrated in vacuo to get 4.73 g (80% yield) of 2-hydroxy-5-(tert-butyl)benzaldehyde as a pale yellow oil. A second component that appeared to contain a minor impurity by TLC was concentrated in vacuo after which the LC showed this to be >96 % pure desired product. This second component amounted to 0.55 g of a pale yellow oil. Total yield was 5.28 g (90% yield)

^1^H NMR (500 MHz, DMSO-d_6_): δ 1.263 (s, 9H), 6.946 (d, 1H), 7.597 (dd, 1H), 7.644 (d, 1H), 10.241 (s, 1H), 10.526 (br s, 1H).

**Step 2: 2-Hydroxy-5-(tert-butyl)benzoic acid:** The following procedure was adapted from that described in WO2005110996A1 [2]: To a flask charged with 2-hydroxy-5-(tert-butyl)benzaldehyde (2.0 g, 0.011 mol) was added 2-methyl-2-butene (55 mL, 0.11 mol) as a 2M solution in THF. Tert-butanol (55 mL) was added. While vigorously stirring the resulting mixture, a solution of sodium dihydrogen phosphate (4.08 g, 0.034 mol) and sodium chlorite (2.08 g, 0.023 mol) in 22 mL of water was added dropwise. After the addition was complete, the mixture was stirred at ambient temperature for 3 h. The mixture was diluted with ethyl acetate (200 mL) and washed with 1N aqueous HCl (200 mL). The aqueous phase was back extracted three times with ethyl acetate (150 mL), and the combined organic phase was washed with a saturated aqueous solution of sodium thiosulfate (200 mL) and brine. The solution was dried over MgSO_4_, filtered, and concentrated in vacuo to give 2.4 g of 2-hydroxy-5-(tert-butyl)benzoic acid as an off-white solid.

MS: m/z 193.1 (MH^-^).

^1^H NMR (500 MHz, DMSO-d_6_): δ 1.295 (s, 9H), 6.891 (d, 1H), 7.574 (dd, 1H), 7.744 (d, 1H), 11.093 (br s, 1H), 13.751 (br s, 1H). NMR indicated the presence of a small amount of 2-methyl-2-butene and other hydrocarbon impurities. This material was used without further purification.

**Step 3:** **Methyl 4-[(5-tert-butyl-2-hydroxybenzene)amido]-3-chlorobenzoate**:

A mixture of 2-hydroxy-5-(tert-butyl)benzoic acid (0.47 g, 2.4 mmol) and methyl 4-amino-3-chlorobenzoate (0.45 g, 2.4mmol) in xylenes (15 mL) was heated to 100^o^C. PCl_3_ (0.54 mL, 1.1 mmol) as a 2M solution in dichloromethane was added dropwise via syringe. The mixture was heated at 124^o^C for 18 h. The temperature was reduced to 100^o^C and the hot solution was pipetted into an Erlenmeyer flask and vigorously stirred while cooling to ambient temperature. The mixture was then stirred in an ice-bath for 45 min. The resulting solid was filtered, washed several times with hexanes and air dried to give 0.46 g of crude product as a white solid. The solid was dissolved in an ethyl acetate/methanol mixture, silica gel was added and the mixture was concentrated in vacuo. The residue was transferred to a pre-column and purified by flash chromatography using a gradient as follows: Hexanes to 30% Ethyl Acetate/hexanes over a 13 min period, then 30% Ethyl Acetate/hexanes for the remainder of the purification. The fractions containing the pure major component were combined and concentrated in vacuo to give 0.38 g (39%) of methyl 4-[(5-tert-butyl-2-hydroxybenzene)amido]-3-chlorobenzoate as a white solid.

MS: m/z 360.1 (MH^-^) and m/z 362.1 (MH^+^).

^1^H NMR (500 MHz, DMSO-d_6_): δ 1.287 (s, 9H), 3.64 (s, 3H), 7.010 (d, 1H), 7.531 (dd, 1H), 7.970 (dd, 1H), 8.045 (br s, 1H), 8.050 (br d, 1H), 8.727 (d, 1H), 11.233 (s, 1H), 11.826 (br s, 1H).

**500200: 4-[(5-tert-butyl-2-hydroxybenzene)amido]-3-chlorobenzoic acid:**

To a mixture of methyl 4-[(5-tert-butyl-2-hydroxybenzene)amido]-3-chlorobenzoate (0.22 g, 0.61 mmol) in ethanol (7.3 mL) at ambient temperature was added 1M aqueous sodium hydroxide (7.3 mL, 7.3 mmol) in one portion. The resulting solution was stirred at ambient temperature for 4 h. The pH was adjusted to ~1 with 1N aqueous hydrogen chloride. The mixture was placed on a rotary evaporator to remove the ethanol. Ethyl acetate and water were added to the aqueous residue and the phases were separated. The aqueous phase was extracted several times with ethyl acetate. The combined organic phase was washed with water then brine and dried over sodium sulfate. The mixture was filtered and the filtrate was concentrated in vacuo to give a quantitative yield of 4-[(5-tert-butyl-2-hydroxybenzene) amido]-3-chlorobenzoic acid as a white solid.

MS: m/z 346 (MH^-^) and m/z 348 (MH^+^).

^1^H NMR (500 MHz, DMSO-d_6_): δ 1.288 (s, 9H), 7.008 (d, 1H), 7.529 (dd, 1H), 7.945 (dd, 1H), 8.019 (d, 1H), 8.051 (d, 1H), 8.691 (d, 1H), 11.207 (s, 1H), 11.812 (br s, 1H), 13.117 (br s, 1H).

**500873:** **4-[(5-tert-butyl-2-hydroxy)amido]-3-(trifluoromethyl)benzoic acid:**

**Step 1: Methyl 4-amino-3-(trifluoromethyl)benzoate:** Thionyl chloride (1.43 g, 0.012 mol) was added dropwise to methanol (8 mL) chilled to -0.6^o^ C at a rate that did not allow the temperature to exceed 0.7^o^ C. The resulting mixture was stirred at 0^o^ C for 45 min., then 4-amino-3-(trifluoromethyl) benzoic acid (0.5 g, 0.0024 mol) was added in one portion. The reaction mixture was stirred at 0^o^ C for one hr., allowed to come to ambient temperature, and stirred for ~3 days. The mixture was concentrated in vacuo. Water (10 mL) was added to the residue and sodium bicarbonate (0.3 g, 3.6 mmol) was added in one portion and the mixture was stirred at ambient temperature for 45 min. The mixture was extracted several times with ethyl acetate. The combined organic phase was dried over sodium sulfate, filtered, and concentrated in vacuo to give 0.49 g (93%) of methyl 4-amino-3-(trifluoromethyl)benzoate as a beige colored solid.

MS: m/z 220.1 (MH^+^).

^1^H NMR (500 MHz, DMSO-d_6_): δ 3.762 (s, 3H), 6.469 (s, 2H), 6.847 (d, 1H), 7.803 (dd, 1H), 7.882 (d, 1H).

**Step 2: 500867: Methyl 4-[(5-tert-butyl-2-hydroxy)amido]-3-(trifluoromethyl)benzoate:** A mixture of 2-hydroxy-5-(tert-butyl)benzoic acid (0.3 g, 1.5 mmol) [**500199, Step 2**] and methyl 4-amino-3-(trifluoromethyl)benzoate (0.33 g, 1.5 mmol) in xylenes (10 mL) was heated to 100^o^C. PCl_3_ (0.4 mL, 0.8 mmol) as a 2M solution in dichloromethane was added dropwise via syringe. The mixture was heated at 130^o^C for 4.5 hr. The temperature was reduced to 100^o^C and the hot solution was pipetted into an Erlenmeyer flask and vigorously stirred while cooling to ambient temperature overnight. The mixture was diluted with ethyl acetate and washed with 1N aqueous hydrogen chloride, saturated aqueous sodium bicarbonate and brine. The reaction mixture was dried over sodium sulfate, filtered, silica gel was added and the filtrate was concentrated in vacuo. The residue was transferred to a pre-column and purified by flash chromatography using a gradient as follows: initially hexanes for one min., then modified the eluent to 10% ethyl acetate/hexanes over a 19 min. period and kept the eluent at 10% ethyl acetate/hexanes for the remainder of the purification. The fractions containing the pure major component were combined and concentrated in vacuo to give 41.8 mg (13.3%) of methyl 4-[(5-tert-butyl-2-hydroxy)amido]-3-(trifluoromethyl)benzoate as an off-white solid.

MS: m/z 394.1 (MH^-^) and m/z 396.1 (MH^+^).

^1^H NMR (500 MHz, DMSO-d_6_): δ 1.286 (s, 9H), 3.898 (s, 3H), 7.000 (d, 1H), 7.538 (dd, 1H), 8.045 (d, 1H), 8.221 (d, 1H), 8.268 (dd, 1H), 8.647 (d, 1H), 11.154 (s, 1H), 11.825 (s, 1H).

**Step 3: 4-[(5-tert-Butyl-2-hydroxy)amido]-3-(trifluoromethyl)benzoic acid:**

To a mixture of methyl 4-[(5-tert-butyl-2-hydroxy)amido]-3-(trifluoromethyl)benzoate (0.11 g, 0.28 mmol) in ethanol (3.4 mL) at ambient temperature was added 1M aqueous sodium hydroxide (3.4 mL, 3.4 mmol) in one portion. The resulting solution was stirred at ambient temperature for 4.5 h. The pH was adjusted to ~8 with 1N aqueous hydrogen chloride (4.4 ml, 4.4 mmol) . The mixture was placed on a rotary evaporator to remove the ethanol. Ethyl acetate and water were added to the aqueous residue and the phases were separated. The aqueous phase was extracted several times with ethyl acetate. The combined organic phase was washed with water, then brine and dried over sodium sulfate. The mixture was filtered and the filtrate was concentrated in vacuo to give 101.5 mg (95.8 % yield) of 4-[(5-tert-butyl-2-hydroxy)amido]-3-(trifluoromethyl)benzoic acid as a white solid.

MS: m/z 380.1 (MH^-^) and m/z 382.1 (MH^+^).

^1^H NMR (500 MHz, DMSO-d_6_): δ 1.344 (s, 9H), 7.056 (d, 1H), 7.593 (dd, 1H), 8.103 (d, 1H), 8.270 (d,1H), 8.298 (dd, 1H), 8.655 (d, 1H), 11.180 (s, 1H), 11.866 (br s, 1H), 13.386 (br s, 1H).

**600494: 3-{[2-Hydroxy-5-(tert-butyl)benzene]amido}-4chloro-benzoic acid:**

**Step 1: 600493: Methyl 3-{[2-hydroxy-5-(tert-butyl)benzene]amido}-4-chloro-benzoate:** A mixture of 2-hydroxy-5-(tert-butyl)benzoic acid (0.25 g, 1.29 mmol) [**500199, Step 2**] and methyl 3-amino-4-chloro-benzoate (0.22g, 1.19 mmol) in xylenes (10 mL) was heated to 100^o^C. PCl_3_ (0.33 mL, 0.65 mmol) as a 2M solution in dichloromethane was added dropwise via syringe. The mixture was heated at 130^o^C for 4 h. The temperature was reduced to 100^o^C and the hot solution was pipetted into an Erlenmeyer flask and vigorously stirred while cooling to ambient temperature overnight. The mixture was diluted to 50 mL with ethyl acetate and washed with 1N aqueous hydrogen chloride (2 X 30 mL), saturated aqueous sodium bicarbonate (2 X 30 mL) and brine. The reaction mixture was dried over sodium sulfate, filtered, and concentrated in vacuo. The residue was dissolved in ethyl acetate, silica gel was added and the mixture was concentrated in vacuo. The residue was transferred to a pre-column and purified by flash chromatography using a gradient as follows: initially hexanes for four min., then modified the eluent to 10% ethyl acetate/hexanes over a 20 min. period. The fractions containing the pure major component were combined and concentrated in vacuo to give 168 mg (39%) of methyl 3-{[2-hydroxy-5-(tert-butyl)benzene]amido}-4-chloro-benzoate as a white solid.

LCMS: 98% by LC/UV; ES+/- m/z 362 [M+H]+, 360 [M-H]-.

^1^H NMR (500 MHz, DMSO-d_6_): δ 1.293 (s, 9H), 3.891 (s, 3H), 7.003 (d, 1H), 7.522 (dd, 1H), 7.722 (d, 2H), 8.061 (d, 1H), 9.136 (t, 1H), 11.091 (s, 1H), 11.778 (s, 1H).

**Step 2: 3-{[2-Hydroxy-5-(tert-butyl)benzene]amido}-4-chlorobenzoic acid:**

To a mixture of Methyl 3-{[2-hydroxy-5-(tert-butyl)benzene]amido}-4-chloro-benzoate (84 mgs, 0.24 mmol) in ethanol (2 mL) at ambient temperature was added 1M aqueous sodium hydroxide (3.0 mL, 2.9 mmol) in one portion. The resulting solution was stirred at ambient temperature for 3 h. The mixture was placed on a rotary evaporator to remove the ethanol. The aqueous residue was treated with 1N aqueous hydrogen chloride (3 mL). Ethyl acetate was added to the mixture, and the phases were separated. The aqueous phase was extracted several times with ethyl acetate. The combined organic phase was washed with brine and dried over sodium sulfate. The mixture was filtered and the filtrate was concentrated in vacuo to give 72 mg (87% yield) of 3-{[2-Hydroxy-5-(tert-butyl)benzene]amido}-4-chlorobenzoic acid as a white solid.

LCMS: 95% by LC/UV; ES+/- m/z 348 [M+H] and 346 [M-H]-.

^1^H NMR (500 MHz, DMSO-d_6_): δ 1.293 (s, 9H), 6.997 (d, 1H), 7.518 (dd, 1H), 7.697 (m, 2H), 8.059 (d, 1H), 9.080 (d, 1H), 11.093 (s, 1H), 11.761 (bs, 1H), 13.2 (bs, 1H).

**600453: 3-Chloro-4-[(3, 5-di-tert-butyl-2-hydroxybenzene)amido]benzoic acid:**

**Step 1: Methyl 3,5-di-tert-butylsalicylate and Methyl 5-tert-butylsalicylate:** This compound was prepared as previously described [3] with some modifications as follows: To a solution of methyl salicylate (5.0g, 0.033 mol) and tert-butanol (6.2g, 0.083 mol) in methanol (3.3 mL) at -3.4^o^C was added slowly dropwise concentrated sulfuric acid (11.6 mL) at such a rate as to keep the temperature below 7^o^C. The resulting mixture was stirred at ambient temperature for 5 h. No solid formed as described in the literature. Water (250 mL) was added to the reaction mixture and the mixture was stirred at ambient temperature for 2.5 days. A white gooey material surrounding the stir bar was obtained. The aqueous solution was decanted and the gooey material was washed twice with water decanting off the aqueous phase each time. Tthe gooey material was dissolved in ethyl acetate and washed with brine, then dried over sodium sulfate. The mixture was filtered and concentrated in vacuo. The residue was dissolved in ethyl acetate, silica gel was added and the mixture was concentrated in vacuo. The residue was transferred to a pre-column and purified by chromatography using a 24g column and hexanes as eluent. Obtained three components: **Component 1** was obtained by combining the fractions containing the pure faster running material and concentrating in vacuo to obtain 4.39g of a colorless oil. **Component 2** was obtained by combining the fractions containing the pure slower running material and concentrating in vacuo to give 0.29g of a colorless oil. **Component 3** was obtained by combining the fractions containing both materials and concentrating in vacuo to give 2.09g of a colorless oil. **Component 3** was rechromatographed as described above. The fractions containing the pure faster running material was combined with **Component 1** above and concentrated in vacuo to give 5.5g (63.2%) methyl 3, 5-di-tert-butylsalicylate as a colorless oil that solidified on cooling to a white solid.

MS: m/z 265.1(MH^+^).

^1^H NMR (500 MHz, CDCl_3_): δ 1.305 (s, 9H), 1.425 (s, 9H), 3.936 (s, 3H), 7.521 (d, 1H), 7.706 (d, 1H), 11.338 (s, 1H).

The fractions containing the slower running material were combined with **Component 2** above and concentrated in vacuo to obtain 0.97 g of methyl 5-tert-butylsalicylate as a colorless oil.

MS: m/z 209.1 (MH^+^).

^1^H NMR (500 MHz, CDCl_3_): δ 1.302 (s, 9H), 3.955 (s, 3H), 6.927 (d, 1H), 7.511 (dd, 1H), 7.818 (d, 1H), 10.592 (s, 1H).

**Step 2: 3, 5-di-tert-Butylsalicylic acid:** This compound was prepared as described in [3] with some modifications as follows: methyl 3, 5-di-tert-butylsalicylate (5.5 g, 0.021 mol) was dissolved in methanol (150 mL). Potassium hydroxide (6.7 g, 0.12 mol) in water (50 mL) was added slowly in a steady stream. The resulting mixture was refluxed for 1h. The hot solution was poured into a 1:1 mixture of ice (200g) and 1N aqueous hydrogen chloride (200 mL), and the mixture was stirred at ambient temperature. The resulting solid was filtered and washed several times with water. The material was air dried to give 4.85g (92% yield) of 3, 5-di-tert-butylsalicylic acid as a white solid.

MS: m/z 249.1 (MH^-^) and m/z 251.1 (MH^+^).

**Step 3: 600452: Methyl 3-chloro-4-[(3, 5-di-tert-butyl-2-hydroxybenzene)amido]benzoate:** A mixture of 3, 5-di-tert-Butylsalicylic acid (0.3 g, 1.1 mmol) and methyl 4-amino-3-chlorobenzoate (0.21 g, 1.1 mmol) in Xylenes (10 mL) was heated to 100^o^C. PCl_3_ (0.28 mL, 0.55 mmol) as a 2M solution in dichloromethane was added dropwise via syringe. The mixture was heated at 130^o^C for 4 hr. The temperature was reduced to 100^o^C and the hot solution was pipetted into an Erlenmeyer flask and vigorously stirred while cooling to ambient temperature overnight. The reaction mixture was diluted with ethyl acetate and washed twice with 1N aqueous hydrogen chloride, twice with aqueous saturated sodium bicarbonate, brine and dried over sodium sulfate. The mixture was filtered, silica gel was added and the mixture was concentrated in vacuo. The residue was transferred to a pre-column and purified by flash chromatography using a gradient as follows: Hexanes for 4 min., then modified the eluent to 5% Ethyl Acetate/hexanes over a 6 min period, then 5% Ethyl Acetate/hexanes for the remainder of the purification. The fractions containing the pure major component were combined and concentrated in vacuo to give 0.20 g (44%) of methyl 3-chloro-4-[(3, 5-di-tert-butyl-2-hydroxybenzene)amido]benzoate as a white solid.

MS: m/z 416.2 (MH^-^) and m/z 418.1 (MH^+^).

^1^H NMR (500 MHz, DMSO-d_6_): 1.323 (s, 9H), 1.392 (s, 9H), 3.896 (s, 3H), 7.482 (d, 1H), 7.735 (d, 1H), 7.896 (d, 1H), 7.994 (dd, 1H), 8.092 (d, 1H), 10.67 (d, 1H), 12.814 (s, 1H).

**Step 4:**  **3-Chloro-4-[(3, 5-di-tert-butyl-2-hydroxybenzene)amido]benzoic acid:**

To a mixture of methyl 3-chloro-4-[(3,5-di-tert-butyl-2-hydroxybenzene)amido]benzoate (0.101 g, 0.24 mmol) in ethanol (2.9 mL) at ambient temperature was added 1M aqueous sodium hydroxide (2.9 mL, 2.9 mmol) in one portion. The resulting solution was stirred at ambient temperature for 5 h. 1N aqueous hydrogen chloride (4 mL) was added and the mixture was stirred at ambient temperature for 15 min. The mixture was placed on a rotary evaporator to remove the ethanol. Ethyl acetate and water were added to the aqueous residue and the phases were separated. The organic phase was washed with water, then brine and dried over sodium sulfate. The mixture was filtered and the filtrate was concentrated in vacuo to give a quantitative yield of 3-chloro-4-[(3,5-di-tert-butyl-2-hydroxybenzene)amido]benzoic acid as a white solid.

MS: m/z 402.1 (MH^-^) and m/z 404.1 (MH^+^).

^1^H NMR (500 MHz, DMSO-d_6_): ^1^H NMR (500 MHz, DMSO-d_6_): 1.324 (s, 9H), 1.393 (s, 9H), 7.479 (d, 1H), 7.697 (d, 1H), 7.899 (d, 1H), 7.967 (dd, 1H), 8.061 (d, 1H), 10.657 (d, 1H), 12.856 (s, 1H), 13.357 (s, 1H).

**600489: Methyl 4-(3-tert-butyl-2-hydroxybenzamido)-3-chlorobenzoate:**

**Step 1: 3-tert-butyl-2-hydroxybenzaldehyde:** To a solution of 2-tert-butylphenol (10.0g, 0.067 mol) in acetonitrile (150 mL) at ambient temperature was added triethylamine (25.4 g, 0.251 mol) followed by MgCl_2_ (9.6 g, 0.10 mol). The resulting mixture was stirred at ambient temperature for 15 min. during which a slight exotherm was observed. Paraformaldehyde (13.6 g, 0.45 mol) was added and the mixture was heated at reflux for 5h. The mixture was allowed cool to ambient temperature and poured into 2N aqueous HCl (200 mL). The mixture was stirred for 30 min. at ambient temperature then extracted several times with diethyl ether. The combined organic phase was washed with brine and dried over MgSO_4_. The mixture was filtered and silica gel was added to the filtrate. This was concentrated in vacuo, transferred to a pre-column, and purified on the Combiflash Rf system using hexane as eluent. The fractions containing the pure major component were combined.

The fractions containing the major component and impurity were combined, silica gel was added and the mixture was concentrated in vacuo then transferred to a pre-column and purified as described above. Combined the fractions containing the pure major component with the fractions from the first purification and concentrated in vacuo to get 6.07 g (51% yield) of 3-tert-butyl-2-hydroxybenzaldehyde as a pale yellow oil.

^1^H NMR (500 MHz, DMSO-d_6_): δ 1.380 (s, 9H), 7.035 (t, 1H), 7.569 (d, 1H), 7.655 (d, 1H), 9.977 (s, 1H), 11.848 (s, 1H).

**Step 2: 3-tert-butylsalicylic acid:** To a flask charged with 3-tert-butyl-2-hydroxybenzaldehyde (2.0 g, 0.011 mol) was added 2-methyl-2-butene (55 mL, 0.11 mol) as a 2M solution in THF. Tert-butanol (55 mL) was added. While vigorously stirring the resulting mixture, a solution of sodium dihydrogen phosphate (4.1 g, 0.034 mol) and sodium chlorite (2.1 g, 0.023 mol) in 34 mL of water was added dropwise. After the addition was complete, the mixture was stirred at ambient temperature for 1.5h. An additional 2.1 g (0.017 mol) of sodium dihydrogen phosphate and 1.1 g (0.012 mol) of sodium chlorite in 17 mL of water was added dropwise and the mixture was stirred at ambient temperature overnight. The mixture was diluted with ethyl acetate (200 mL) and washed twice with 1N aqueous HCl (150 mL). The aqueous phase was back extracted with ethyl acetate and the combined organic phase was washed twice with a saturated aqueous solution of sodium thiosulfate (100 mL), then brine. The solution was dried over MgSO_4_, filtered and concentrated in vacuo. The residue was treated with hexanes and concentrated in vacuo. Treatment with hexanes was repeated several more times, each time concentrating in vacuo to give 2.76g of crude 3-tert-butylsalicylic acid as a gooey tan solid. The gooey tan solid was dissolved in saturated aqueous sodium bicarbonate solution and the solution was extracted with dichloromethane. The aqueous phase was filtered and the pH was adjusted to ~2 with concentrated hydrochloric acid. The resulting solid was filtered, washed several times with water and air dried to give 1.58 g (73.8%) of 3-tert-butylsalicylic acid as a white solid.

MS of the crude product: m/z 193.1 (MH^-^).

^1^H NMR (500 MHz, DMSO-d_6_): δ 1.374 (s, 9H), 6.850 (t, 1H), 7.464 (d, 1H), 7.697 (d, 1H), 12.231 (br s, 1H), ~13.9 (br s, 1H).

**Step 3: Methyl 4-(3-tert-butyl-2-hydroxybenzamido)-3-chlorobenzoate:**

A mixture of 3-tert-butylsalicylic acid (0.25g, 1.3 mmol) and methyl 4-amino-3-chlorobenzoate (0.24g, 1.3 mmol) in xylenes (15 mL) was heated to 100^o^C. PCl_3_ (0.33 mL, 0.65 mmol) as a 2M solution in dichloromethane was added dropwise via syringe. The mixture was heated at 130^o^C for 2.75 h. The temperature was reduced to 100^o^C and the hot solution was pipetted into an Erlenmeyer flask and vigorously stirred while cooling to ambient temperature overnight. The reaction mixture was diluted with ethyl acetate and washed with 2 X saturated aqueous sodium bicarbonate, 2 X 1N aqueous hydrogen chloride and brine. The organic phase was dried over sodium sulfate, filtered and concentrated in vacuo. The residue was dissolved in ethyl acetate, silica gel was added and the mixture was concentrated in vacuo. The residue was transferred to a pre-column and purified by chromatography using a gradient as follows: initially hexanes for 2 min., then the eluent was modified to 10% ethyl acetate/hexanes over a 10 min. period and kept at 10% ethyl acetate/hexanes for the remainder of the separation. The fractions containing the pure desired (by LC/MS) component were combined and concentrated in vacuo to give a 116.2 mg (24.7% yield) of methyl 4-(3-tert-butyl-2-hydroxybenzamido)-3-chlorobenzoate as a white solid.

MS: m/z 360 (MH^-^) and m/z 362 (MH^+^).

^1^H NMR (500 MHz, DMSO-d_6_): δ 1.387 (s, 9H), 3.893 (s, 3H), 6.931(t, 1H), 7.478 (dd, 1H), 7.740 (d, 1H), 7.941 (dd, 1H), 7.985 (dd, 1H), 8.080 (d, 1H), 10.629 (s, 1H), 12.963 (s, 1H).

**600653: Methyl 4-(2-hydroxy-5-[trifluoromethyl]benzamido)-3-bromobenzoate:**

**Step 1: 2-Hydroxy-5-(trifluoromethyl)benzaldehyde:** The following procedure was adapted from that described[1]: To a solution of 4-trifluoromethylphenol (5.0 g, 0.31 mol) in acetonitrile (140 mL) at ambient temperature was added triethylamine (11.4 g, 0.113 mol) followed by MgCl_2_ (4.5 g, 0.47 mol). The resulting mixture was stirred at ambient temperature for 15 min. during which a slight exotherm was observed. Paraformaldehyde (6.2 g, 0.206 mol) was added and the mixture was heated at reflux for 3h then allowed to cool to ambient temperature and poured the mixture into 10% aqueous HCl (260 mL). The mixture was stirred for 30 min. at ambient temperature, then extracted several times with diethyl ether. The combined organic phase was washed with brine and dried over MgSO_4._ The mixture was filtered and silica gel was added to the filtrate. This was concentrated in vacuo, transferred to a pre-column, and purified by chromatography using a gradient as follows: initially hexanes (20 min.), then the eluent was modified to 10% EtOAc/hexanes over 10 min. and kept at 10% EtOAc/hexanes for the remainder of the purification. The fractions containing the pure fastest eluting main component were combined and concentrated in vacuo to get 1.77 g (30% yield) of 2-hydroxy-5-(trifluoromethyl)benzaldehyde as a white solid.

MS: m/z 189 (MH^-^).

^1^H NMR (500 MHz, DMSO-d_6_): δ 7.196 (d, 1H), 7.848 (dd, 1H), 7.922 (br d, 1H), 10.312 (s, 1H), 11.555 (br s, 1H).

**Step 2: 2-Hydroxy-5-(trifluoromethyl)benzoic acid:** The following procedure was adapted from that described in WO2005110996A1 [2]: To a flask charged with 2-hydroxy-5-(trifluoromethyl)benzaldehyde (1.6 g, 0.008 mol) was added 2-methyl-2-butene (40 mL, 0.08 mol) as a 2M solution in THF. Tert-butanol (40 mL) was added. While vigorously stirring the resulting mixture, a solution of sodium dihydrogen phosphate (3.0 g, 0.025 mol) and sodium chlorite (1.53 g, 0.017 mol) in 16 mL of water was added dropwise. After the addition was complete, the mixture was stirred at ambient temperature for 2.5 h. The mixture was diluted with ethyl acetate (150 mL) and washed with 1N aqueous HCl (200 mL). The aqueous phase was back extracted with ethyl acetate and the combined organic phase was washed with a saturated aqueous solution of sodium thiosulfate (150 mL) then brine. The solution was dried over MgSO_4_, filtered and concentrated in vacuo to give 2.02 g of 2-hydroxy-5-(trifluoromethyl)benzoic acid as a white solid.

MS: m/z 205 (MH^-^).

^1^H NMR (500 MHz, DMSO-d_6_): δ 7.146 (d, 1H), 7.827 (dd, 1H), 8.089 (br d, 1H), 11.109 (br s, 1H), 13.671 (br s, 1H). NMR indicated the presence of a small amount of the aldehyde and 2-methyl-2-butene. This material was used without further purification.

**Step 3: Methyl 4-(2-hydroxy-5-[trifluoromethyl]benzamido)-3-bromobenzoate:**

A mixture of 2-hydroxy-5-(trifluoromethyl)benzoic acid (0.20 g, 1.0 mmol) and methyl 4-amino-3-bromobenzoate (0.22 g, 1.0mmol) in xylenes (10 mL) was heated to 100^o^C. PCl_3_ (0.25 mL, 0.50 mmol) as a 2M solution in dichloromethane was added dropwise via syringe. The mixture was heated at 130^o^C for 4.5 hr. The temperature was reduced to 100^o^C and the hot solution was pipetted into an Erlenmeyer flask and vigorously stirred while cooling to ambient temperature overnight. The reaction mixture was diluted with ethyl acetate and washed twice with saturated aqueous sodium bicarbonate, twice with 1M aqueous sodium bisulfate and brine. The organic phase was dried over sodium sulfate, filtered, silica gel was added and the mixture was concentrated in vacuo. The residue was transferred to a pre-column and purified by chromatography using a gradient as follows: initially hexanes for 4 min., then the eluent was modified to 25% ethyl acetate/hexanes over a 10 min. period and kept at 25% ethyl acetate/hexanes for the remainder of the separation. The fractions containing the pure desired (by LC/MS) component were combined and concentrated in vacuo to give a 161.3 mg (38.6% yield) of methyl 4-(2-hydroxy-5-[trifluoromethyl]benzamido)-3-bromobenzoate as a white solid.

MS: m/z 415.8 (MH^-^) and m/z 417.8 (MH^+^).

^1^H NMR (500 MHz, DMSO-d_6_): δ 3.857 (s, 3H), 7.247 (d, 1H), 7.828 (dd, 1H), 8.012 (dd, 1H), 8.199 (d, 1H), 8.305 (d, 1H), 8.628 (d, 1H), 11.048 (s, 1H), 13.011 (br s, 1H).

**600655: Methyl 4-(2-hydroxy-5-[trifluoromethyl]benzamido)-3-methylbenzoate:**

A mixture of 2-hydroxy-5-(trifluoromethyl)benzoic acid (0.20 g, 1.0 mmol)[from **600653, Step 2**] and methyl 4-amino-3-methylbenzoate (0.17 g, 1.0mmol) in xylenes (10 mL) was heated to 100^o^C. PCl_3_ (0.25 mL, 0.50 mmol) as a 2M solution in dichloromethane was added dropwise via syringe. The mixture was heated at 130^o^C for 4.5 hr. The temperature was reduced to 100^o^C and the hot solution was pipetted into an Erlenmeyer flask and vigorously stirred while cooling to ambient temperature overnight. The resulting solid was filtered and washed twice with ice-cold xylenes, then several times with hexanes and air dried overnight. The solid was dissolved in ethyl acetate, silica gel was added and the mixture was concentrated in vacuo. The residue was transferred to a pre-column and purified by chromatography using a gradient as follows: initially hexanes for 4 min., then the eluent was modified to 20% ethyl acetate/ hexane over a 10 min. period and kept at 20% ethyl acetate/hexane for the remainder of the separation. The fractions containing the desired (by LC/MS) component and impurity were combined and concentrated in vacuo. The residue was dissolved in ethyl acetate and washed with 1M aqueous sodium bisulfate and brine. The organic phase was dried over sodium sulfate, filtered, concentrated in vacuo. Sample is still impure. The residue was dissolved in ethyl acetate, silica gel was added and the mixture was concentrated in vacuo. The residue was transferred to a pre-column and purified by chromatography using a gradient as follows: initially hexanes for 4 min., then the eluent was modified to 10% ethyl acetate/hexanes over a 6 min. period and kept at 10% ethyl acetate/hexane for 14 min. Finally, modified eluent to 15% ethyl acetate/hexanes over 2 min period and kept the eluent at 15% ethyl acetate/hexanes for the remainder of the separation. The fractions containing the pure desired product were combined and concentrated in vacuo to give a 133 mg (37.7% yield) of methyl 4-(2-hydroxy-5-[trifluoromethyl] benzamido)-3-methylbenzoate as an off- white solid.

MS: m/z 352 (MH^-^) and m/z 354 (MH^+^).

^1^H NMR (500 MHz, DMSO-d_6_): δ 2.336 (s, 3H), 3.794 (s, 3H), 7.181 (d, 1H), 7.755 (dd, 1H), 7.805 (dd, 1H), 7.839 (s, 1H), 8.240 (m, 2H), 10.510 (s, 1H).

**600657:** **4-(2-Hydroxy-5-methoxybenzamido)-3-methylbenzoic Acid:**

**Step 1:600656 Methyl 4-(2-hydroxy-5-methoxybenzamido)-3-methylbenzoate:** A mixture of 2-hydroxy-5-methoxybenzoic acid (0.20 g, 1.2 mmol) and methyl 4-amino-3-methyl benzoate (0.20 g, 1.2 mmol) in xylenes (10 mL) was heated to 100^o^C. PCl_3_ (0.3 mL, 0.60 mmol) as a 2M solution in dichloromethane was added dropwise via syringe. The mixture was heated at 130^o^C for 6 hr. The temperature was reduced to 100^o^C and the hot solution was pipetted into an Erlenmeyer flask. The hot solution was stirred vigorously while cooling to ambient temperature overnight. The resulting solid was filtered, washed twice with ice-cold xylenes, several times with hexanes and air dried to give 251 mg (66.4% yield) of methyl 4-(2-hydroxy-5-methoxybenzamido)-3-methylbenzoate as a light turquoise solid.

MS: m/z 314 (MH^-^) and m/z 316 (MH^+^).

^1^H NMR (500 MHz, DMSO-d_6_): δ 2.365 (s, 3H), 3.748 (s, 3H), 3.832 (s, 3H), 6.983 (d, 1H), 7.071 (dd, 1H), 7.542 (d, 1H), 7.838 (dd, 1H), 7.875 (d, 1H), 8.348 (d, 1H), 10.662 (s, 1H), 11.523 (br s, 1H).

**Step 2:** **4-(2-Hydroxy-5-methoxybenzamido)-3-methylbenzoic Acid:**

To a mixture of methyl 4-(2-hydroxy-5-methoxybenzamido)-3-methylbenzoate (0.11 g, 0.35 mmol) in ethanol (4.2 mL) at ambient temperature was added 1M aqueous sodium hydroxide (4.2 mL, 4.2 mmol) in one portion. The resulting solution was stirred at ambient temperature for 24 h. 1N aqueous hydrogen chloride (6 mL) was added and the mixture was stirred at ambient temperature for 30 min. The mixture was placed on a rotary evaporator to remove the ethanol. Ethyl acetate and water were added to the aqueous residue and the phases were separated. The aqueous phase was extracted with ethyl acetate. The combined organic phase was washed with water, then brine and dried over sodium sulfate. The mixture was filtered and the filtrate was concentrated in vacuo to give 94.7 mg (89.9% yield) of 4-(2-hydroxy-5-methoxybenzamido)-3-methylbenzoic acid as an off-white solid.

MS: m/z 300 (MH^-^) and m/z 302 (MH^+^).

^1^H NMR (500 MHz, DMSO-d_6_): δ 2.427 (s, 3H), 3.820 (s, 3H), 7.052 (d, 1H), 7.145 (dd, 1H) 7.618 (d, 1H), 7.882 (dd, 1H), 7.923 (d, 1H), 8.363 (d, 1H), 10.706 (s, 1H), 11.594 (s, 1H), 12.799 (br s, 1H).

**500189: Methyl 3-chloro-4-[(5-chloro-2-hydroxybenzene)amido]benzoate:**

A mixture of 5-chloro-2-hydroxybenzoic acid (0.50 g, 2.7 mmol) and methyl 4-amino-3-chorobenzoate (0.47 g, 2.7 mmol) in xylenes (15 mL) was heated to 115^o^C. PCl_3_ (0.54 mL, 1.0 mmol) as a 2M solution in dichloromethane was added dropwise via syringe. The mixture was heated at reflux for 4 h. The temperature was reduced to 100^o^C and the hot solution was pipetted into an Erlenmeyer flask and vigorously stirred while cooling to ambient temperature. The resulting solid was filtered and washed several times with hexanes. The solid was placed in ethyl acetate (10 mL) and heated to boiling. MeOH was added one milliliter at a time for a total of 16 mL. Some solid remained. The hot solution was filtered and the solid was labeled as component A and air dried overnight to get 157 mg. A crystalline material began forming in the filtrate. Hexanes (15 mL) were added and the flask was stored at ambient temperature overnight. The resulting solid was filtered and air dried to give 155 mg. Labeled this solid component B. Both components gave the same results by LC/MS and NMR. Total yield was 312 mg (34% yield) of methyl 3-chloro-4-[(5-chloro-2-hydroxybenzene)amido]benzoate as a white solid.

MS: m/z 338 (MH^-^) and m/z 340 (MH^+^).

^1^H NMR (500 MHz, DMSO-d_6_): δ 3.866 (s, 3H), 7.097 (d, 1H), 7.531 (dd, 1H), 7.981 (m, 2H), 8.051 (br d, 1H), 8.690 (d, 1H), 11.117 (s, 1H), 12.398 (s, 1H).

**500844: Methyl 3-chloro-4-{[2-hydroxy-5-(trifluoromethyl)benzene]amido}benzoate:**

A mixture of 5-trifluoromethylsalicylic acid (0.49 g, 2.4 mmol) [**600653, Step 2**] and methyl 4-amino-3-chlorobenzoate (0.45 g, 2.4 mmol) in xylenes (15 mL) was heated to 100^o^C. PCl_3_ (0.54 mL, 1.1 mmol) as a 2M solution in dichloromethane was added dropwise via syringe. The mixture was heated at 130^o^C for 5 h. The temperature was reduced to 100^o^C and the hot solution was pipetted into an Erlenmeyer flask. The hot solution was stirred vigorously while cooling to ambient temperature. The resulting solid was filtered, washed several times with hexane and air dried to get 359 mg of crude product as a white solid. The solid was dissolved in ethyl acetate, silica gel was added and the mixture was concentrated in vacuo. The residue was transferred to a pre-column and purified by chromatography using a gradient as follows: initially hexanes (1 min.), then the eluent was modified to 30% ethyl acetate/hexane over a 12 min. period and then kept at 30% ethyl acetate/hexane for the remainder of the purification. The fractions containing the pure major component were combined and concentrated in vacuo to give 297 mg (33%) of methyl 3-chloro-4-{[2-hydroxy-5-(trifluoromethyl)benzene]amido}benzoate as a white solid.

MS: m/z 372 (MH^-^).

^1^H NMR (500 MHz, DMSO-d_6_): δ 3.873 (s, 3H), 7.257 (d, 1H), 7.841 (dd, 1H), 7.996 (dd, 1H), 8.066 (d, 1H), 8.317 (br d, 1H), 8.697 (d, 1H), 11.179 (s, 1H), 13.036 (br s, 1H).

**500828: Methyl 3-chloro-4-[(2-hydroxy-5-methylbenzene)amido]benzoate:**

A mixture of 5-methylsalicylic acid (0.30 g, 2.0 mmol) and methyl 4-amino-3-chlorobenzoate (0.37 g, 2.0 mmol) in Xylenes (10 mL) was heated to 124^o^C. PCl_3_ (0.40 mL, 0.8 mmol) as a 2M solution in dichloromethane was added dropwise via syringe. The mixture was heated at reflux for 4 h. The temperature was reduced to 100^o^C and the hot solution was pipetted into an Erlenmeyer flask and vigorously stirred while cooling to ambient temperature over an hour. The resulting solid was filtered, washed several times with hexanes and air dried to get 430 mg of crude product as a white solid. The solid was dissolved in ethyl acetate, silica gel was added and the mixture was concentrated in vacuo. The residue was transferred to a pre-column and purified by chromatography using a gradient as follows: initially hexanes to 30% ethyl acetate/hexanes over a 6 min. period and then kept at 30% ethyl acetate/hexane for the remainder of the purification. The fractions containing the pure major component were combined and concentrated in vacuo to give 286 mg (45% yield) of methyl 3-chloro-4-[(2-hydroxy-5-methylbenzene)amido]benzoate as a white solid.

MS: m/z 318(MH^-^) and m/z 320 (MH^+^).

^1^H NMR (500 MHz, DMSO-d_6_): δ 2.284 (s, 3H), 3.864 (s, 3H), 6.968 (d, 1H), 7.288 (dd, 1H), 7.844 (d, 1H), 7.974(dd, 1H), 8.045 (d, 1H), 8.722 (d, 1H), 11.243 (br s, 1H), 11.786 (br s, 1H).

Supporting Information References:

1. Knight PD, O'Shaughnessy PN, Munslow IJ, Kimberley BS, Scott P. Biaryl-bridged Schiff base complexes of zirconium alkyls: synthesis structure and stability. Journal of Organometallic Chemistry. 2003;683(1):103-13. doi: <https://doi.org/10.1016/S0022-328X(03)00455-8>.

2. Fotouhi NH, Gregory Jay; Simonsen, Klaus B.; Vu, Binh Thanh; Webber, Stephen Evan, inventorPreparation of novel optically active cis-​1-​carbamoyl-​2,​4,​5-​triphenyl-​2-​imidazolines as inhibitors of interaction of MDM2 protein with p53-​like peptide useful against solid tumors. U.S2005 10.05.2005.

3. Jiménez CA, Belmar JB. Convenient and Efficient Method for the Obtainment of Ketones from Highly Hindered Aromatic N,N‐Dimethyl‐amides. Synthetic Communications. 2007;37(14):2391-7. doi: 10.1080/00397910701411077.
