# Supplementary figures and images for: N-substituted phenylbenzamides of the niclosamide chemotype attenuate obesity related changes in high fat diet fed mice
Source: PLoS One. 2018 Oct 25;13(10):e0204605. doi: 10.1371/journal.pone.0204605 (PMC6201879; doi:10.1371/journal.pone.0204605)

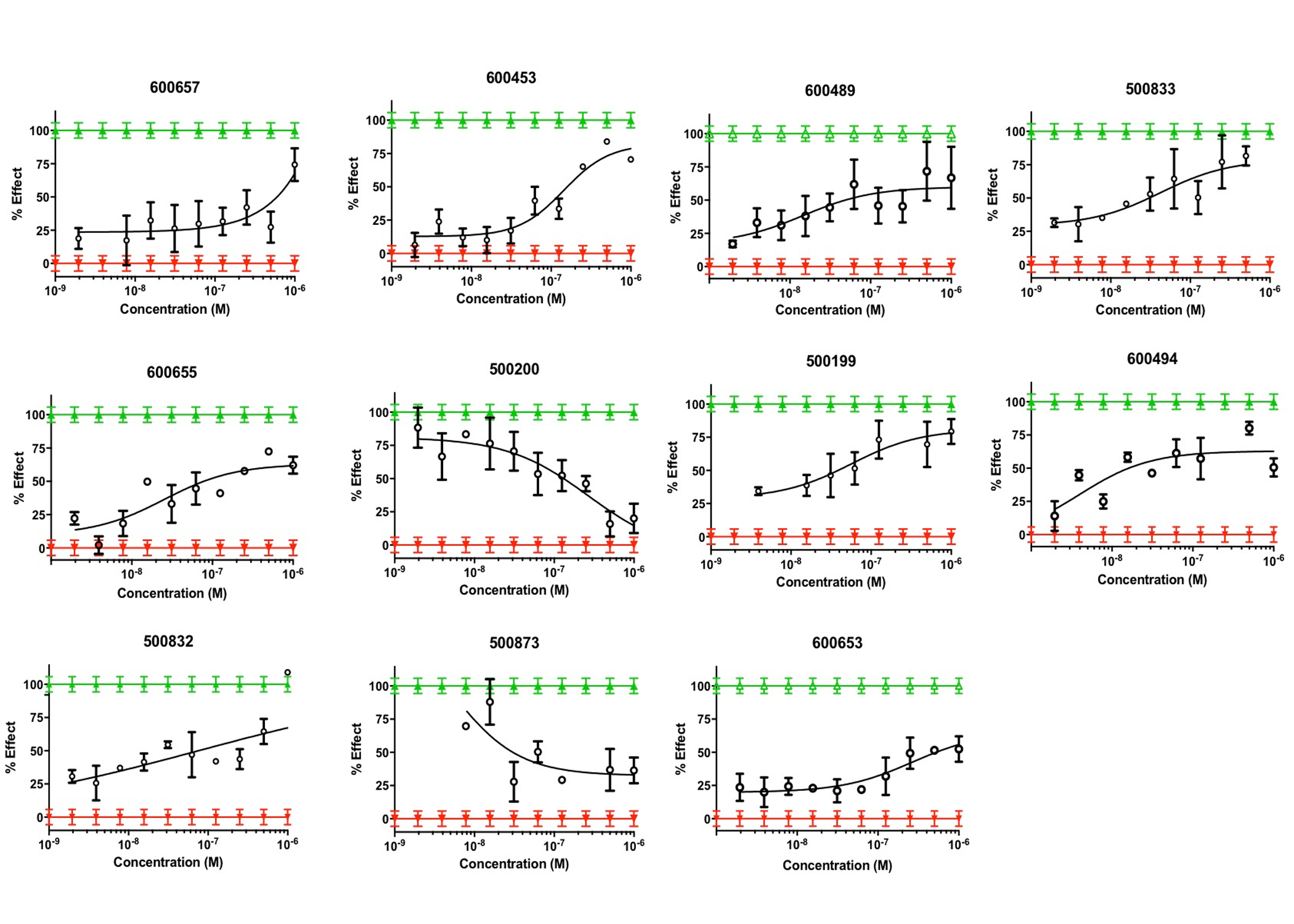

Supplement: S1 Fig — (TIF) [file pone.0204605.s003.tif]
